# Design, Synthesis, and Evaluation of a New Series of Hydrazones as Small-Molecule Akt Inhibitors for NSCLC Therapy

**DOI:** 10.1021/acsomega.3c02331

**Published:** 2023-05-24

**Authors:** Burak Erdönmez, Mehlika Dilek Altıntop, Gülşen Akalın Çiftçi, Ahmet Özdemir, Abdulilah Ece

**Affiliations:** † Department of Pharmaceutical Chemistry, Graduate School of Health Sciences, Anadolu University, 26470 Eskişehir, Turkey; ‡ Department of Pharmaceutical Chemistry, Faculty of Pharmacy, Anadolu University, 26470 Eskişehir, Turkey; § Department of Biochemistry, Faculty of Pharmacy, Anadolu University, 26470 Eskişehir, Turkey; ∥ Department of Pharmaceutical Chemistry, Faculty of Pharmacy, Biruni University, 34010 Istanbul, Turkey

## Abstract

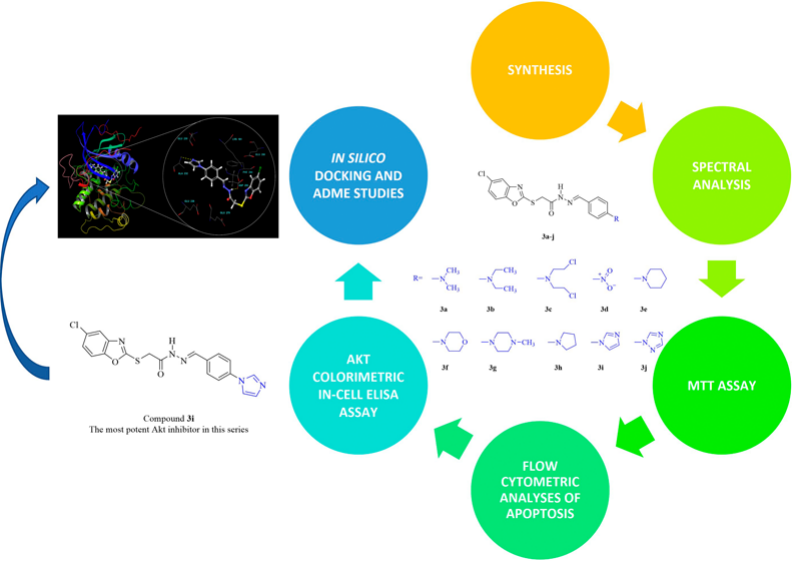

In an endeavor to identify small molecules for the management of non-small-cell lung carcinoma, 10 new hydrazone derivatives (**3a–j**) were synthesized. MTT test was conducted to examine their cytotoxic activities against human lung adenocarcinoma (A549) and mouse embryonic fibroblast (L929) cells. Compounds **3a**, **3e**, **3g**, and **3i** were determined as selective antitumor agents on A549 cell line. Further studies were conducted to figure out their mode of action. Compounds **3a** and **3g** markedly induced apoptosis in A549 cells. However, both compounds did not show any significant inhibitory effect on Akt. On the other hand, *in vitro* experiments suggest that compounds **3e** and **3i** are potential anti-NSCLC agents acting through Akt inhibition. Furthermore, molecular docking studies revealed a unique binding mode for compound **3i** (the strongest Akt inhibitor in this series), which interacts with both hinge region and acidic pocket of Akt2. However, it is understood that compounds **3a** and **3g** exert their cytotoxic and apoptotic effects on A549 cells *via* different pathway(s).

## 1 Introduction

Cancer remains a deadly global health concern with more than 200 different types, affecting over 60 human organs.^1^ Among all cancers, lung cancer is the primary cause of cancer-related morbidity and mortality in males, while, in females, it ranks second for mortality, after breast cancer, and third for incidence, after breast and colorectal cancer.^2^ The disease is notorious for its exceptional potency to spread to distant parts of the body (metastasis), as well as its ability to progress rapidly in its early stages.^3^

There are two groups of lung cancer, namely, small cell lung carcinoma (SCLC) and non-small-cell lung carcinoma (NSCLC).^1^ 85% of lung cancer cases are attributed to NSCLC.^4^

Surgery, chemotherapy, radiotherapy, immunotherapy, and targeted therapy are currently available approaches for NSCLC therapy.^5^ The best treatment strategy for patients with early-stage NSCLC is still surgical intervention. However, surgery is no longer an option for patients with advanced or metastatic NSCLC.^6,7^ Major treatment approaches for unresectable NSCLC are chemotherapy and radiotherapy.^7^ Platinum-based chemotherapeutics, taxanes, and other chemotherapeutics (e.g., vinorelbine, gemcitabine, and pemetrexed) are used either alone or in combination.^5^ Despite their benefits in NSCLC therapy, these chemotherapeutics damage healthy cells as well as cancer cells and therefore they cause severe adverse effects and toxicity.^8^ Resistance to radio(chemo)therapy is also a significant barrier to NSCLC therapy resulting in tumor recurrence and disease progression.^9^ The current challenges in the diagnosis and treatment of NSCLC lead to poor prognosis and low survival rate.^10^

Akt belongs to the family of serine/threonine-specific protein kinases essential for regulating crucial cellular processes including cell survival, proliferation, growth, apoptosis, and glucose metabolism. The abnormal overexpression or activation of Akt is involved in a variety of human malignancies and therefore inhibiting Akt has emerged as a pivotal strategy for the treatment of various types of cancer, particularly B-cell malignancies, NSCLC, and breast cancer.^11−13^ Despite the large number of Akt inhibitors developed to date, the U.S. Food and Drug Administration has not approved any Akt inhibitors yet.^11^

Hydrazides-hydrazones are frequently occurring eligible motifs in druglike small molecules due to their distinctive characteristics and several pharmaceutical applications for the treatment of many diseases, particularly severe bacterial infections, cancer, and inflammation.^14−18^ Hydrazones have been reported to exert striking antitumor action *via* induction of apoptosis, cell cycle arrest, inhibition of angiogenesis, and a plethora of cancer-related biological targets (e.g., Akt).^19−26^

Benzoxazoles are privileged building blocks for the synthesis of biologically active ligands targeting a plethora of crucial targets/pathways due to their unique features allowing them to effectively bind to diverse biological targets with distinct affinities.^27−31^ Several studies have revealed that benzoxazoles exert marked cytotoxic activity against a variety of cancer cell lines through diverse mechanisms including induction of apoptosis, inhibition of Akt phosphorylation, and so on.^32−39^

**Figure 1:**
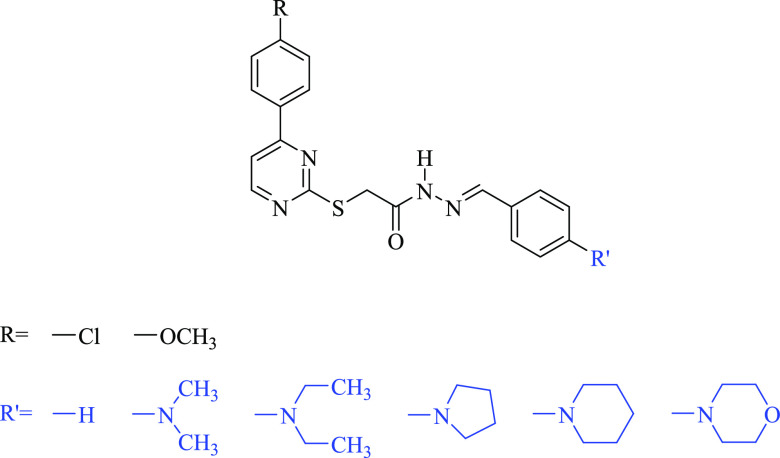
Pyrimidine-based hydrazones reported previously as anticancer agents by our research team.^21^

The publications related to hydrazones^19−26^ and benzoxazoles^32−39^ exerting pronounced anticancer activity through inhibition of target enzymes involved in the pathogenesis of lung cancer motivated us to design novel anti-NSCLC agents by means of the molecular hybridization of the benzoxazole core with the hydrazide group, which was conjugated with the benzylidene moiety substituted at the *para* position with dialkylamino groups (dimethylamino, diethylamino), nitrogen-containing electron-withdrawing groups (nitro substituent), five- (pyrrolidine) or six-membered heterocyclic motifs (piperidine, morpholine and piperazine), heteroaromatic rings (imidazole, triazole) based on our previous work (Figure 1),^21^ and the structure–activity relationships of existing Akt inhibitors^11^ together with the bioisosteric replacement. In this context, the designed compounds were synthesized readily and assessed for their cytotoxic properties on human lung adenocarcinoma (A549) and mouse embryonic fibroblast (L929) cells. *In vitro* experimental studies were carried out for promising anti-NSCLC agents to shed light on their mechanism of action.

## 2 Results and Discussion

### 2.1 Chemistry

**Scheme 1:**
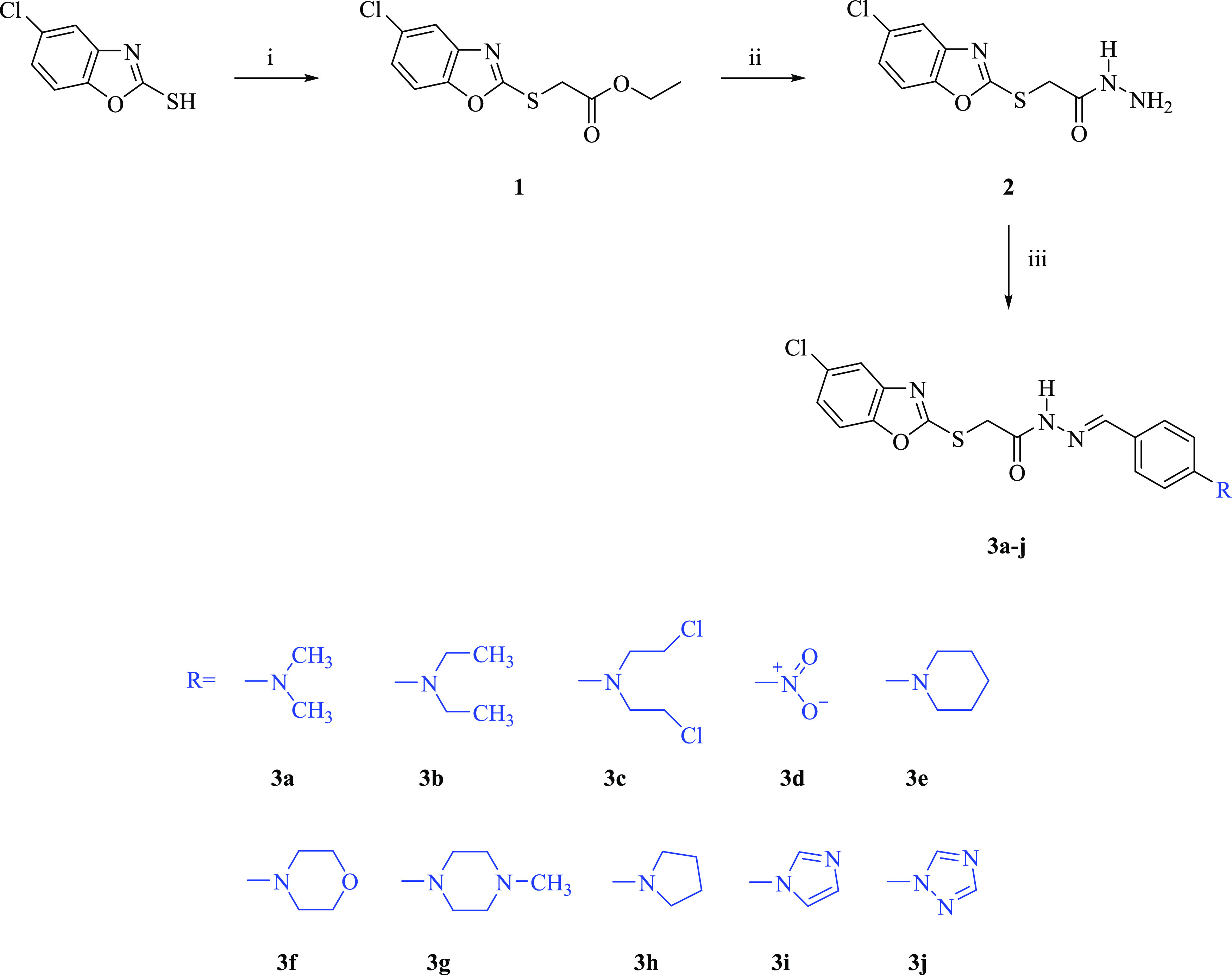
Synthesis of Compounds **3a–j** (i) ClCH_2_COOEt, K_2_CO_3_, acetone, reflux, 10 h; (ii) NH_2_NH_2_.H_2_O, ethanol, stirring at room temperature, 5 h; (iii) ArCHO, ethanol, reflux, 8 h.

The hitherto unreported hydrazones (**3a**–**j**) were prepared as depicted in Scheme 1. The base-catalyzed reaction of 5-chloro-2-mercaptobenzoxazole with ethyl chloroacetate yielded ethyl 2-[(5-chlorobenzoxazol-2-yl)thio]acetate (**1**), which subsequently underwent a reaction with hydrazine hydrate affording the corresponding hydrazide (**2**). Finally, compound **2** was reacted with 4-substituted benzaldehydes to obtain compounds **3a**–**j**. Infrared (IR), nuclear magnetic resonance (NMR, ^1^H and ^13^C), and high-resolution mass spectrometry (HRMS) were used to verify their chemical structures. These spectra are provided in the Supporting Information.

In the IR spectra of compounds **3a–j**, the N–H stretching band of the hydrazone group was detected at 3207.62–3159.40 cm^–1^, while the C=O stretching band was detected at 1705.07–1654.92 cm^–1^. In the ^1^H NMR spectra of compounds **3a–j**, S-CH_2_ protons gave rise to two singlets in the range of 4.25–4.73 ppm. The peaks at 11.47–12.09 ppm were attributed to the N–H proton of the hydrazone moiety, while the peaks in the region 7.88–8.26 ppm were assigned to the CH=N proton. In the ^13^C NMR spectra of compounds **3a–j**, the S-CH_2_ carbon gave rise to a singlet in the region 34.76–35.02 ppm. The signals due to the CH=N and the C=O carbons were detected in the range of 142.95–145.19 ppm and 167.08–168.27 ppm, respectively. In all HRMS spectra, the predicted and measured *m*/*z* values for [M + H]^+^ were consistent with each other.

### 2.2 Biochemistry

The hydrazide (**2**) and the hydrazones (**3a–j**) were assessed for their cytotoxic features on A549 human lung adenocarcinoma and L929 mouse embryonic fibroblast cells. The IC_50_ values of the compounds are presented in Table 1. Compounds **3a**, **3e**, **3g**, and **3i** showed cytotoxic effects on A549 cells with IC_50_ values of 91.35 ± 21.89, 176.23 ± 56.50, 46.60 ± 6.15, and 83.59 ± 7.30 μM, respectively. The selectivity index (SI) values of compounds **3a**, **3e**, **3g**, and **3i** were found as >5.47, >2.84, 5.94, and >5.98, respectively. It can be concluded that these agents exert cytotoxic activity against A549 cells without influencing normal (L929) cells at their effective doses.

**Table 1:** IC_50_ and SI Data for Compounds **2**, **3a–j**, and Cisplatin

compound	IC_50_ (μM)		SI^a^
	A549 cell line	L929 cell line	
**2**	>500	>500	
**3a**	91.35 ± 21.89	>500	>5.47
**3b**	410.15 ± 220.38	>500	
**3c**	>500	>500	
**3d**	>500	>500	
**3e**	176.23 ± 56.50	>500	>2.84
**3f**	250.61 ± 45.87	>500	
**3g**	46.60 ± 6.15	276.67 ± 28.87	5.94
**3h**	>500	>500	
**3i**	83.59 ± 7.30	>500	>5.98
**3j**	>500	>500	
**Cisplatin**	23.89 ± 2.71		

a SI = IC_50_ for L929 cells/IC_50_ for A549 cells.

According to the data presented in Table 1, the replacement of the dimethylamino group (compound **3a**) with the diethylamino substituent (compound **3b**) led to a significant decline in anticancer activity. This outcome indicated that the elongation of the alkyl chains reduced the cytotoxic effect on A549 cells. The bis(2-chloroethyl) and the nitro groups caused the loss of anticancer activity.

Among the six-membered heterocyclic rings (piperidine, morpholine, and piperazine) attached to the 4th position of the benzylidene motif, the piperazine ring gave rise to a significant increase in anticancer activity against A549 cells. On the contrary, the introduction of the pyrrolidine scaffold into the 4th position of the benzylidene group (compound **3h**) resulted in the loss of anticancer activity. Taking into account the IC_50_ values of compounds **3i** and **3j** (Table 1), the 1*H*-imidazole core increased the cytotoxic effect on A549 cell line, while the 1*H*-1,2,4-triazole ring caused the loss of anticancer activity.

**Figure 2:**
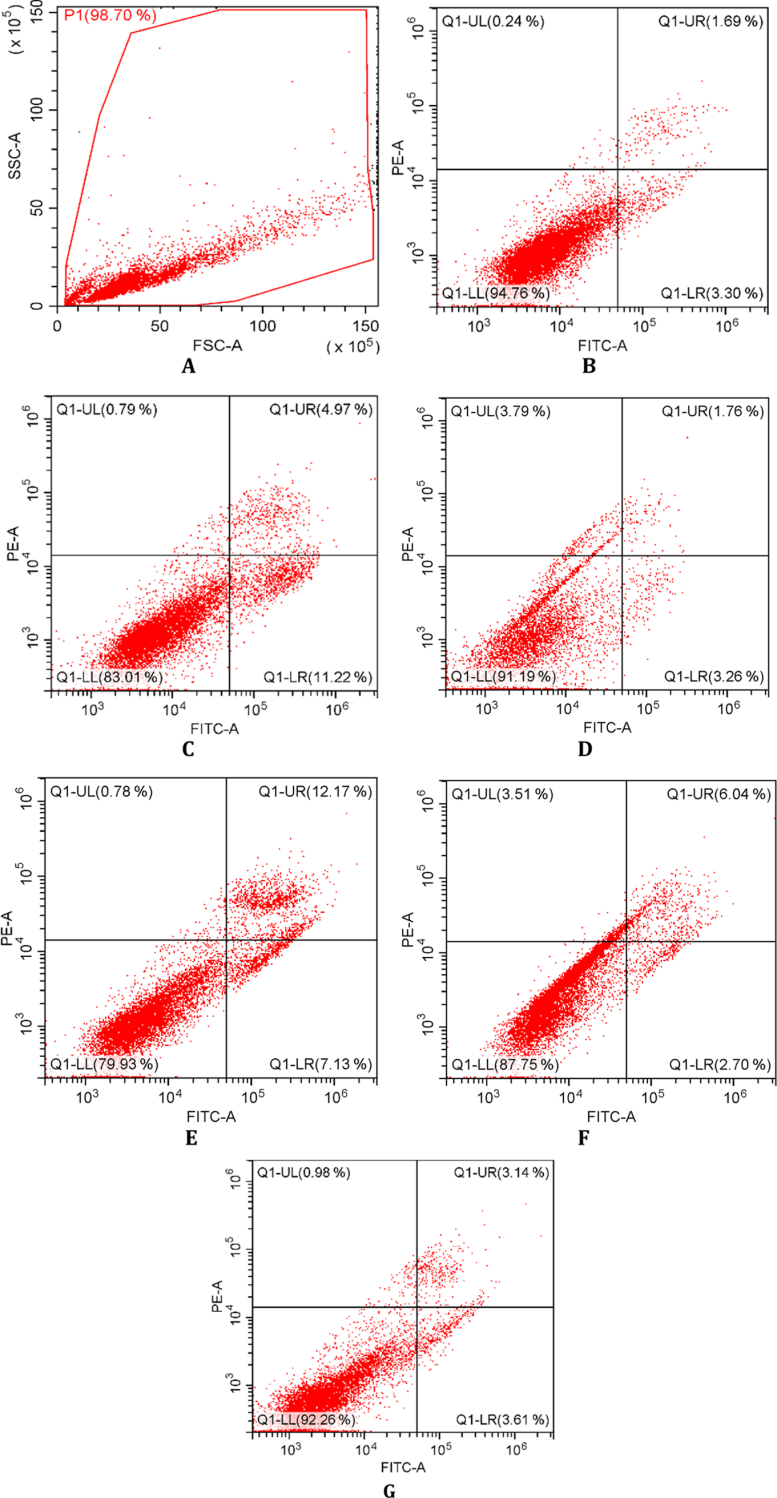
Flow cytometric analysis of A549 cells treated with IC_50_ values of compounds **3a**, **3e**, **3g**, **3i**, and cisplatin. At least 10,000 cells were analyzed per sample, and quadrant analysis was performed. Q1-UL, Q1-LL, Q1-UR, and Q1-LR quadrants represent necrosis, viability, and late and early apoptosis, respectively. (A) The main gate selected from the cell population, (B) control, (C) compound **3a**, (D) compound **3e**, (E) compound **3g**, (F) compound **3i**, and (G) cisplatin.

Further studies were carried out for compounds **3a**, **3e**, **3g**, and **3i** to illuminate their mechanism of anti-NSCLC action. In this context, after incubation of A549 cells exposed to these agents and cisplatin for 24 h, flow cytometry-based apoptosis detection assay was performed. The percentages of A549 cells undergoing early apoptosis caused by compounds **3a**, **3e**, **3g**, **3i**, and cisplatin (at 91.35, 176.23, 46.60, 83.59, and 23.89 μM, respectively) were found to be 11.22, 3.26, 7.13, 2.70, and 3.61%, respectively (Table 2, Figure 2). The percentages of late apoptotic cells induced by compounds **3a**, **3e**, **3g**, **3i**, and cisplatin were determined as 4.97, 1.76, 12.17, 6.04, and 3.14%, respectively. In particular, compounds **3a** and **3g** showed more apoptotic activity than cisplatin in A549 cells.

**Table 2:** Percents of Typical Quadrant Analysis of Annexin V Fluorescein Isothiocyanate (FITC)/PI Flow Cytometry of A549 Cells Exposed to Compounds **3a**, **3e**, **3g**, **3i**, and Cisplatin

groups	early apoptosis (%)	late apoptosis (%)	necrosis (%)	viability (%)
control	3.30	1.69	0.24	94.76
cells treated with compound **3a**	11.22	4.97	0.79	83.01
cells treated with compound **3e**	3.26	1.76	3.79	91.19
cells treated with compound **3g**	7.13	12.17	0.78	79.93
cells treated with compound **3i**	2.70	6.04	3.51	87.75
cells treated with **cisplatin**	3.61	3.14	0.98	92.26

A colorimetric method was used to assess the inhibitory effects of compounds **3a**, **3e**, **3g**, and **3i** on Akt in A549 cells. Compound **3e** carrying a piperidine ring in the 4th position of the benzylidene motif and compound **3i** bearing an imidazole ring in the 4th position of the benzylidene moiety inhibited Akt in A549 cells with IC_50_ values of 105.88 ± 53.71 and 69.45 ± 1.48 μM, respectively, compared to GSK690693 (IC_50_ = 5.93 ± 1.20 μM), a well-known Akt inhibitor. According to the data indicated in Table 3, it can be concluded that compounds **3e** and **3i** exert their anti-NSCLC action through the inhibition of Akt.

**Table 3:** Akt Inhibitory Effects of Compounds **3a**, **3e**, **3g**, **3i**, GSK690693, and Cisplatin in A549 Cell Line

compound	IC_50_ (μM)
**3a**	
**3e**	105.88 ± 53.71
**3g**	
**3i**	69.45 ± 1.48
**GSK690693**	5.93 ± 1.20
**Cisplatin**	8.09 ± 0.63

Dimethylamino-substituted compound **3a** caused 35.40 ± 13.36% Akt inhibition at 91.35 μM. Compound **3g**, which carries the 4-methylpiperazine motif in the 4th position of the benzylidene moiety, did not exert any inhibitory effect on Akt. Accordingly, it is obvious that both compounds cause cytotoxicity and apoptosis in A549 cell line *via* different pathway(s).

### 2.3 *In Silico* Studies

Computer-aided drug design tools provide a detailed picture of the biologically active molecules at the molecular level. Our recent successful *in silico* studies^40−42^ prompted us to confer molecular docking simulations as a tool to enlighten binding conformations of the ligands.

Before moving forward to *in silico* calculations, internal validation^43^ was carried out to test how well our docking method performs. The conformation of co-crystallized ligand (GSK690693) was successfully reproduced with Glide XP method yielding a root-mean-square deviation (RMSD) of 0.436.

**Figure 3:**
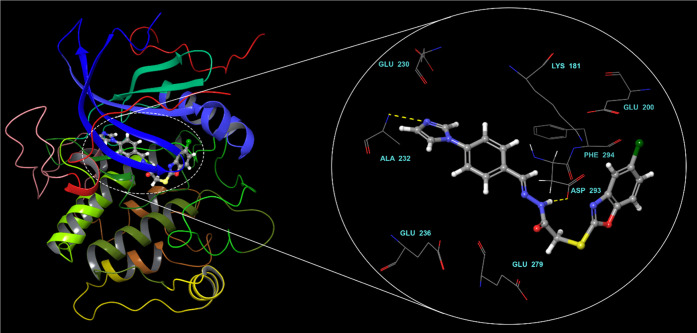
Binding mode of compound **3i** within the binding region of Akt2.

Molecular docking studies showed the binding mode of compound **3i** within Akt2 binding site (Figure 3). The imidazole nitrogen engages in a key hydrogen bond with the backbone amine of ALA232 in the hinge region. The sulfur linker seems to facilitate compound **3i** to adopt a unique binding mode. This folded mode that resembles a V-shaped conformation brings the benzoxazole ring to the vicinity of the acidic pocket. A new hydrogen bond is observed between ASP293 and the N–H of the hydrazide spacer. The interaction of a ligand with this acidic pocket was reported to be important for potent inhibition.^44^

Certain descriptors related to absorption, distribution, metabolism, and excretion (ADME) were also predicted. Blood–brain barrier parameter was also calculated that could be useful in central nervous system-targeted studies. Of note is that compounds **3a**, **3e**, **3g**, and **3i** were estimated to have good pharmacokinetic profiles and druglike properties (Table 4).

**Table 4:** Predicted ADME Properties of Compounds **3a**, **3e**, **3g**, and **3i**^a^

compound	MW	QPlogPo/w	QPlogBB^b^	HOA%^c^	PSA^d^	rule of five^e^
**3a**	388.87	4.65	–0.77	100.00	76.85	0.00
**3e**	428.94	5.41	–0.79	100.00	78.08	1.00
**3g**	443.95	4.09	–0.26	94.58	82.42	0.00
**3i**	411.87	4.54	–1.06	100.00	89.18	0.00

a Octanol/water partition coefficient (recommended range: −2.0 to 6.5).

b Brain/blood partition coefficient (recommended range: −3.0 to 1.2).

c Human oral absorption (HOA) (<25% is poor, >80% is high).

d Polar surface area (PSA) (recommended range: 7.0–200.0).

e Number of violations of Lipinski’s rule of five.

## 3 Conclusions

This paper describes the synthesis of new hydrazones (**3a–j**) designed as small molecules for NSCLC therapy. Taking into account *in vitro* cytotoxicity data, selective anticancer agents in this series on A549 cells were determined as compounds **3a** (IC_50_ = 91.35 ± 21.89 μM), **3e** (IC_50_ = 176.23 ± 56.50 μM), **3g** (IC_50_ = 46.60 ± 6.15 μM), and **3i** (IC_50_ = 83.59 ± 7.30 μM). Among them, compounds **3a** and **3g** caused induction of apoptosis in A549 cells stronger than cisplatin. Both compounds did not exert any significant Akt inhibitory activity in A549 cells, while compounds **3e** and **3i** caused Akt inhibition in A549 cells. It can be concluded that compounds **3e** and **3i** exert their cytotoxic action against A549 cell line *via* the inhibition of Akt. Molecular docking simulations disclosed a unique binding mode for compound **3i** (the most active Akt inhibitor in this series) which interacts with the hinge region and the acidic pocket of Akt2. On the other hand, further research is required to shed light on the mechanism of action underlying the cytotoxic and apoptotic effects of compounds **3a** and **3g** on A549 cells.

## 4 Materials and Methods

### 4.1 Chemistry

The chemicals were procured from Acros Organics (Geel, Belgium), Maybridge (Loughborough, UK), Merck (Darmstadt, Germany), and Sigma-Aldrich (St. Louis, MO, USA). Melting points (M.p.) were determined by an Electrothermal IA9200 digital melting point apparatus (Staffordshire, U.K.). IR spectra were acquired from an IRPrestige-21 Fourier Transform (FT)-IR spectrophotometer (Shimadzu, Tokyo, Japan). ^1^H and ^13^C NMR spectra were recorded on a Varian Mercury-400 FT-NMR spectrometer (Agilent, Palo Alto, CA, USA). HRMS spectra were acquired from an LCMS-IT-TOF (Shimadzu, Kyoto, Japan) using the electrospray ionization (ESI) technique. Thin-layer chromatography (TLC) was employed to track the progress of all chemical reactions and examine the purity of the synthesized agents.

#### 4.1.1 Synthesis of Ethyl 2-[(5-chlorobenzoxazol-2-yl)thio]acetate (**1**)

A mixture of 5-chloro-2-mercaptobenzoxazole (0.06 mol), potassium carbonate (0.06 mol), and ethyl chloroacetate (0.06 mol) in acetone (60 mL) was heated under reflux for 10 h. The precipitated ester was filtered off and washed with distilled water. After drying, the product was crystallized from ethanol.

Yield: 98%, M.p.: 65–67 °C, Lit. M.p.: 64–65 °C.^45^ IR ν_max_ (cm^–1^): 3091.89, 3068.75, 3022.45, 2978.09, 2931.80, 1735.93, 1612.49, 1496.76, 1462.04, 1446.61, 1429.25, 1375.25, 1309.67, 1257.59, 1234.44, 1219.01, 1193.94, 1174.65, 1141.86, 1109.07, 1058.92, 1026.13, 937.40, 920.05, 898.83, 867.97, 860.25, 819.75, 767.67, 715.59, 702.09, 677.01. ^1^H NMR (400 MHz, DMSO-*d*_6_) δ (ppm): 1.19 (t, *J* = 7.0 Hz, 3H), 4.16 (q, *J* = 7.2 Hz, 2H), 4.30 (s, 2H), 7.36 (dd, *J* = 8.4 Hz, 2.0 Hz, 1H), 7.68 (d, *J* = 9.2 Hz, 1H), 7.74 (d, *J* = 2.4 Hz, 1H). ^13^C NMR (100 MHz, DMSO-*d*_6_) δ (ppm): 13.97 (CH_3_), 33.78 (CH_2_), 61.57 (CH_2_), 111.54 (CH), 118.12 (CH), 124.34 (CH), 129.01 (C), 142.37 (C), 150.15 (C), 165.45 (C), 167.69 (C). HRMS (ESI) (*m*/*z*): [M + H]^+^ calcd for C_11_H_10_ClNO_3_S: 272.0143, found, 272.0141.

#### 4.1.2 Synthesis of 2-[(5-Chlorobenzoxazol-2-yl)thio]acetohydrazide (**2**)

A mixture of compound **1** (0.05 mol) and hydrazine hydrate (0.10 mol) was stirred in ethanol for 5 h at room temperature. The precipitated hydrazide was filtered off and washed with ethanol. After drying, it was crystallized from ethanol.

Yield: 66%, M.p.: 159–161 °C, Lit. M.p.: 190–192 °C.^45^ IR ν_max_ (cm^–1^): 3284.77, 3157.47, 3068.75, 2999.31, 2953.02, 2873.94, 1651.07, 1629.85, 1548.84, 1494.83, 1465.90, 1448.54, 1423.47, 1406.11, 1338.60, 1253.73, 1238.30, 1211.30, 1136.07, 1105.21, 1051.20, 1010.70, 956.69, 933.55, 918.12, 864.11, 821.68, 806.25, 773.46, 729.09, 686.66. ^1^H NMR (400 MHz, DMSO-*d*_6_) δ (ppm): 3.35 (brs, 1H), 4.10 (s, 2H), 4.40 (brs, 1H), 7.35 (dd, *J* = 8.6 Hz, 2.2 Hz, 1H), 7.67 (d, *J* = 8.8 Hz, 1H), 7.72 (d, *J* = 2.4 Hz, 1H), 8.86 and 9.44 (2brs, 1H). ^13^C NMR (100 MHz, DMSO-*d*_6_) δ (ppm): 34.06 (CH_2_), 111.50 (CH), 117.99 (CH), 124.24 (CH), 128.93 (C), 142.50 (C), 150.09 (C), 165.45 (C), 165.87 (C). HRMS (ESI) (*m*/*z*): [M + H]^+^ calcd for C_9_H_8_ClN_3_O_2_S: 258.0099, found, 258.0109.

#### 4.1.3 General Procedure for the Preparation of *N*′-(4-Subsittuted benzylidene)-2-[(5-chlorobenzoxazol-2-yl)thio]acetohydrazide (**3a–j**)

A mixture of compound **2** (0.0012 mol) and 4-substituted benzaldehyde (0.0012 mol) was heated under reflux in ethanol for 8 h. The precipitate was filtered off and washed with ethanol. After drying, the product was crystallized from ethanol.

##### 4.1.3.1 *N*′-(4-Dimethylaminobenzylidene)-2-[(5-chlorobenzoxazol-2-yl)thio]acetohydrazide (**3a**)

Yield: 62%, M.p.: 197–199 °C. IR ν_max_ (cm^–1^): 3167.12, 3074.53, 3018.60, 2951.09, 2889.37, 1666.50, 1602.85, 1535.34, 1492.90, 1456.26, 1442.75, 1402.25, 1365.60, 1317.38, 1249.87, 1230.58, 1209.37, 1178.51, 1159.22, 1134.14, 1101.35, 1051.20, 948.98, 937.40, 918.12, 854.47, 819.75, 808.17, 796.60, 786.96, 702.09, 669.30. ^1^H NMR (400 MHz, DMSO-*d*_6_) δ (ppm): 2.96 (s, 6H), 4.25 and 4.65 (2s, 2H), 6.71 (d, *J* = 8.8 Hz, 2H), 7.35 (d, *J* = 8.8 Hz, 2.0 Hz, 1H), 7.50 (d, *J* = 8.8 Hz, 2H), 7.68 (dd, *J* = 8.8 Hz, 2.6 Hz, 1H), 7.75 (dd, *J* = 8.4 Hz, 2.0 Hz, 1H), 7.91 and 8.06 (2s, 1H), 11.52 and 11.56 (2brs, 1H). ^13^C NMR (100 MHz, DMSO-*d*_6_) δ (ppm): 35.02 (CH_2_), 39.64 (2CH_3_), 111.42 (CH), 111.73 (2CH), 118.06 (CH), 121.15 (CH), 124.15 (C), 128.24 (2CH), 128.52 (C), 142.58 (C), 144.96 (CH), 150.10 (C), 151.43 (C), 166.32 (C), 167.19 (C). HRMS (ESI) (*m*/*z*): [M + H]^+^ calcd for C_18_H_17_ClN_4_O_2_S: 389.0834, found, 389.0853.

##### 4.1.3.2 *N*′-(4-Diethylaminobenzylidene)-2-[(5-chlorobenzoxazol-2-yl)thio]acetohydrazide (**3b**)

Yield: 65%, M.p.: 172–173 °C. IR ν_max_ (cm^–1^): 3172.90, 3082.25, 2968.45, 2927.94, 2893.22, 1666.50, 1610.56, 1597.06, 1527.62, 1494.83, 1463.97, 1444.68, 1421.54, 1400.32, 1386.82, 1375.25, 1350.17, 1313.52, 1273.02, 1253.73, 1213.23, 1197.79, 1182.36, 1155.36, 1134.14, 1105.21, 1082.07, 1051.20, 1012.63, 1004.91, 954.76, 929.69, 914.26, 881.47, 860.25, 813.96, 779.24, 758.02, 715.59, 700.16, 677.01. ^1^H NMR (400 MHz, DMSO-*d*_6_) δ (ppm): 1.09 (t, *J* = 7.0 Hz, 6H), 3.34–3.39 (m, 4H), 4.25 and 4.64 (2s, 2H), 6.65 (d, *J* = 8.8 Hz, 2H), 7.35 (dd, *J* = 8.6 Hz, 2.2 Hz, 1H), 7.45 (d, *J* = 8.8 Hz, 2H), 7.69 (dd, *J* = 8.6 Hz, 1.8 Hz, 1H), 7.75 (dd, *J* = 10.0 Hz, 2.0 Hz, 1H), 7.88 and 8.03 (2s, 1H), 11.49 and 11.52 (2s, 1H). ^13^C NMR (100 MHz, DMSO-*d*_6_) δ (ppm): 12.41 (2CH_3_), 35.01 (CH_2_), 43.74 (2CH_2_), 111.02 (2CH), 111.47 (CH), 118.06 (CH), 120.22 (CH), 124.15 (C), 128.57 (2CH), 128.91 (C), 142.59 (C), 145.00 (CH), 148.77 (C), 150.11 (C), 166.33 (C), 167.14 (C). HRMS (ESI) (*m*/*z*): [M + H]^+^ calcd for C_20_H_21_ClN_4_O_2_S: 417.1147, found, 417.1171.

##### 4.1.3.3 *N*′-[4-(Bis(2-chloroethyl)amino)benzylidene]-2-[(5-chlorobenzoxazol-2-yl)thio]acetohydrazide (**3c**)

Yield: 47%, M.p.: 155–156 °C. IR ν_max_ (cm^–1^): 3180.62, 3072.60, 3012.81, 2962.66, 2895.15, 1662.64, 1598.99, 1525.69, 1510.26, 1494.83, 1448.54, 1413.82, 1390.68, 1355.96, 1315.45, 1276.88, 1255.66, 1203.58, 1176.58, 1136.07, 1107.14, 1053.13, 958.62, 929.69, 916.19, 910.40, 864.11, 815.89, 804.32, 746.45, 717.52, 678.94. ^1^H NMR (400 MHz, DMSO-*d*_6_) δ (ppm): 3.74–3.79 (m, 8H), 4.26 and 4.65 (2s, 2H), 6.79 (d, *J* = 9.2 Hz, 2H), 7.35 (dd, *J* = 8.4 Hz, 2.4 Hz, 1H), 7.52 (d, *J* = 9.2 Hz, 2H), 7.68 (dd, *J* = 8.6 Hz, 2.2 Hz, 1H), 7.74 (dd, *J* = 9.0 Hz, 2.2 Hz, 1H), 7.91 and 8.07 (2s, 1H), 11.56 and 11.60 (2s, 1H). ^13^C NMR (100 MHz, DMSO-*d*_6_) δ (ppm): 35.00 (CH_2_), 40.98 (2CH_2_), 51.85 (2CH_2_), 111.46 (CH), 111.76 (2CH), 118.06 (CH), 122.29 (CH), 124.15 (C), 128.59 (2CH), 128.91 (C), 142.56 (C), 144.53 (CH), 147.94 (C), 150.11 (C), 166.28 (C), 167.31 (C). HRMS (ESI) (*m*/*z*): [M + H]^+^ calcd for C_20_H_19_Cl_3_N_4_O_2_S: 485.0367, found, 485.0394.

##### 4.1.3.4 *N*′-(4-Nitrobenzylidene)-2-[(5-chlorobenzoxazol-2-yl)thio]acetohydrazide (**3d**)

Yield: 88%, M.p.: 237–239 °C. IR ν_max_ (cm^–1^): 3201.83, 3093.82, 2960.73, 2846.93, 1680.00, 1614.42, 1597.06, 1585.49, 1516.05, 1487.12, 1452.40, 1404.18, 1379.10, 1336.67, 1255.66, 1226.73, 1201.65, 1151.50, 1139.93, 1112.93, 1105.21, 1064.71, 929.69, 918.12, 898.83, 889.18, 860.25, 850.61, 833.25, 821.68, 804.32, 748.38, 736.81, 704.02, 690.52. ^1^H NMR (400 MHz, DMSO-*d*_6_) δ (ppm): 4.33 and 4.73 (2s, 2H), 7.35 (dd, *J* = 8.6 Hz, 1.8 Hz, 1H), 7.68 (dd, *J* = 8.8 Hz, 4.8 Hz, 1H), 7.73–7.74 (m, 1H), 7.96–7.99 (m, 2H), 8.14 (s, 1H), 8.26–8.32 (m, 2H), 12.09 (brs, 1H). ^13^C NMR (100 MHz, DMSO-*d*_6_) δ (ppm): 34.76 (CH_2_), 111.48 (CH), 118.06 (CH), 124.02 (2CH), 124.21 (CH), 127.89 (2CH), 128.94 (C), 140.14 (C), 142.49 (C), 144.87 (CH), 147.80 (C), 150.11 (C), 166.02 (C), 168.27 (C). HRMS (ESI) (*m*/*z*): [M + H]^+^ calcd for C_16_H_11_ClN_4_O_4_S: 391.0262, found, 391.0278.

##### 4.1.3.5 *N*′-[4-(Piperidin-1-yl)benzylidene]-2-[(5-chlorobenzoxazol-2-yl)thio]acetohydrazide (**3e**)

Yield: 77%, M.p.: 187–188 °C. IR ν_max_ (cm^–1^): 3165.19, 3076.46, 3008.95, 2920.23, 2850.79, 1664.57, 1600.92, 1523.76, 1494.83, 1456.26, 1442.75, 1425.40, 1386.82, 1354.03, 1315.45, 1226.73, 1209.37, 1184.29, 1163.08, 1126.43, 1099.43, 1051.20, 1020.34, 952.84, 935.48, 918.12, 852.54, 806.25, 794.67, 715.59, 702.09, 677.01. ^1^H NMR (400 MHz, DMSO-*d*_6_) δ (ppm): 1.56 (brs, 6H), 3.24 (brs, 4H), 4.26 and 4.65 (2s, 2H), 6.91–6.95 (m, 2H), 7.35 (dd, *J* = 8.8 Hz, 2.0 Hz, 1H), 7.50 (dd, *J* = 8.6 Hz, 5.0 Hz, 2H), 7.68 (dd, *J* = 8.6 Hz, 3.0 Hz, 1H), 7.74 (dd, *J* = 7.8 Hz, 1.8 Hz, 1H), 7.91 and 8.06 (2s, 1H), 11.56 and 11.61 (2s, 1H). ^13^C NMR (100 MHz, DMSO-*d*_6_) δ (ppm): 23.92 (CH_2_), 24.95 (2CH_2_), 34.98 (CH_2_), 48.40 (2CH_2_), 111.47 (CH), 114.58 (2CH), 118.06 (CH), 122.94 (CH), 124.15 (C), 128.18 (2CH), 128.47 (C), 142.57 (C), 144.62 (CH), 150.11 (C), 152.35 (C), 166.28 (C), 167.30 (C). HRMS (ESI) (*m*/*z*): [M + H]^+^ calcd for C_21_H_21_ClN_4_O_2_S: 429.1147, found, 429.1153.

##### 4.1.3.6 *N*′-[4-(Morpholin-4-yl)benzylidene]-2-[(5-chlorobenzoxazol-2-yl)thio]acetohydrazide (**3f**)

Yield: 82%, M.p.: 209–210 °C. IR ν_max_ (cm^–1^): 3180.62, 3078.39, 3010.88, 2962.66, 2854.65, 1666.50, 1604.77, 1523.76, 1498.69, 1446.61, 1425.40, 1400.32, 1381.03, 1311.59, 1269.16, 1253.73, 1228.66, 1211.30, 1184.29, 1166.93, 1132.21, 1122.57, 1111.00, 1101.35, 1051.20, 948.98, 927.76, 918.12, 862.18, 819.75, 808.17, 792.74, 717.52, 702.09, 677.01. ^1^H NMR (400 MHz, DMSO-*d*_6_) δ (ppm): 3.19 (t, *J* = 4.8 Hz, 2H), 3.28 (t, *J* = 4.8 Hz, 2H), 3.71–3.75 (m, 4H), 4.26 and 4.66 (2s, 2H), 6.87 (d, *J* = 8.8 Hz, 2H), 7.35 (dd, *J* = 8.6 Hz, 2.2 Hz, 1H), 7.55 (d, *J* = 8.8 Hz, 2H), 7.69 (dd, *J* = 8.6 Hz, 3.8 Hz, 1H), 7.73 (d, *J* = 8.8 Hz, 1H), 7.94 and 8.10 (2s, 1H), 11.63 (brs, 1H). ^13^C NMR (100 MHz, DMSO-*d*_6_) δ (ppm): 34.91 (CH_2_), 46.90 (2CH_2_), 65.82 (2CH_2_), 111.44 (CH), 113.78 (2CH), 117.51 (CH), 122.25 (CH), 124.55 (C), 128.09 (C), 130.12 (2CH), 142.55 (C), 144.46 (CH), 150.09 (C), 152.16 (C), 166.24 (C), 167.37 (C). HRMS (ESI) (*m*/*z*): [M + H]^+^ calcd for C_20_H_19_ClN_4_O_3_S: 431.0939, found, 431.0954.

##### 4.1.3.7 *N*′-[4-(4-Methylpiperazin-1-yl)benzylidene]-2-[(5-chlorobenzoxazol-2-yl)thio]acetohydrazide (**3g**)

Yield: 35%, M.p.: 178–179 °C. IR ν_max_ (cm^–1^): 3205.69, 3061.03, 2949.16, 2935.66, 2845.00, 2802.57, 1654.92, 1610.56, 1600.92, 1562.34, 1517.98, 1496.76, 1448.54, 1425.40, 1409.96, 1381.03, 1361.74, 1315.45, 1294.24, 1251.80, 1234.44, 1209.37, 1190.08, 1161.15, 1130.29, 1107.14, 1078.21, 1055.06, 999.13, 960.55, 918.12, 875.68, 819.75, 794.67, 700.16, 673.16. ^1^H NMR (400 MHz, DMSO-*d*_6_) δ (ppm): 2.23 (s, 3H), 2.45 (t, *J* = 4.8 Hz, 4H), 3.24 (t, *J* = 4.6 Hz, 4H), 4.28 and 4.67 (2s, 2H), 6.96–6.99 (m, 2H), 7.37 (dd, *J* = 8.6 Hz, 1.8 Hz, 1H), 7.55 (dd, *J* = 9.2 Hz, 4.4 Hz, 2H), 7.70 (dd, *J* = 8.6 Hz, 3.4 Hz, 1H), 7.76 (dd, *J* = 7.4 Hz, 1.8 Hz, 1H), 7.94 and 8.10 (2s, 1H), 11.60 and 11.66 (2s, 1H). ^13^C NMR (100 MHz, DMSO-*d*_6_) δ (ppm): 34.97 (CH_2_), 45.74 (CH_3_), 47.09 (2CH_2_), 54.40 (2CH_2_), 111.47 (CH), 114.50 (2CH), 118.07 (CH), 123.63 (CH), 124.17 (C), 128.16 (2CH), 128.40 (C), 128.91 (C), 142.57 (C), 144.53 (CH), 150.11 (C), 166.28 (C), 167.35 (C). HRMS (ESI) (*m*/*z*): [M + H]^+^ calcd for C_21_H_22_ClN_5_O_2_S: 444.1255, found, 444.1278.

##### 4.1.3.8 *N*′-[4-(Pyrrolidin-1-yl)benzylidene]-2-[(5-chlorobenzoxazol-2-yl)thio]acetohydrazide (**3h**)

Yield: 82%, M.p.: 206–207 °C. IR ν_max_ (cm^–1^): 3170.97, 3080.32, 2976.16, 2956.87, 2096.73, 2850.79, 1666.50, 1612.49, 1598.99, 1529.55, 1494.83, 1448.54, 1436.97, 1386.82, 1348.24, 1315.45, 1301.95, 1249.87, 1217.08, 1178.51, 1163.08, 1138.00, 1107.14, 1049.28, 958.62, 931.62, 916.19, 902.69, 858.32, 810.10, 779.24, 700.16, 675.09. ^1^H NMR (400 MHz, DMSO-*d*_6_) δ (ppm): 1.93–1.98 (m, 4H), 3.26 (t, *J* = 6.6 Hz, 4H), 4.25 and 4.64 (2s, 2H), 6.52–6.56 (m, 2H), 7.35 (dd, *J* = 8.6 Hz, 2.2 Hz, 1H), 7.48 (dd, *J* = 9.0 Hz, 5.0 Hz, 2H), 7.66–7.69 (m, 1H), 7.74 (dd, *J* = 8.4 Hz, 2.0 Hz, 1H), 7.89 and 8.05 (2s, 1H), 11.47 and 11.51 (2s, 1H). ^13^C NMR (100 MHz, DMSO-*d*_6_) δ (ppm): 24.93 (2CH_2_), 34.99 (CH_2_), 47.19 (2CH_2_), 111.42 (CH), 111.49 (2CH), 118.03 (CH), 122.25 (CH), 124.12 (C), 128.36 (2CH), 128.64 (C), 142.57 (C), 145.19 (CH), 148.82 (C), 150.08 (C), 166.30 (C), 167.08 (C). HRMS (ESI) (*m*/*z*): [M + H]^+^ calcd for C_20_H_19_ClN_4_O_2_S: 415.0990, found, 415.1013.

##### 4.1.3.9 *N*′-[4-(1*H*-Imidazol-1-yl)benzylidene]-2-[(5-chlorobenzoxazol-2-yl)thio]acetohydrazide (**3i**)

Yield: 83%, M.p.: 229–231 °C. IR ν_max_ (cm^–1^): 3159.40, 3099.61, 2978.09, 2823.79, 1705.07, 1608.63, 1577.77, 1521.84, 1487.12, 1465.90, 1448.54, 1427.32, 1400.32, 1359.82, 1305.81, 1263.37, 1228.66, 1215.15, 1176.58, 1136.07, 1107.14, 1060.85, 989.48, 962.48, 927.76, 916.19, 866.04, 835.18, 817.82, 719.45, 702.09, 680.87, 651.94. ^1^H NMR (400 MHz, DMSO-*d*_6_) δ (ppm): 4.31 and 4.71 (2s, 2H), 7.14 (brs, 1H), 7.34 (dd, *J* = 8.4 Hz, 2.4 Hz, 1H), 7.68 (dd, *J* = 8.6 Hz, 5.0 Hz, 1H), 7.73–7.76 (m, 3H), 7.82–7.86 (m, 3H), 8.08 and 8.26 (2s, 1H), 8.35 (brs, 1H), 11.86 and 11.92 (2s, 1H). ^13^C NMR (100 MHz, DMSO-*d*_6_) δ (ppm): 34.81 (CH_2_), 111.44 (CH), 117.78 (CH), 118.03 (CH), 120.29 (2CH), 124.17 (CH), 128.38 (2CH), 128.90 (CH), 130.13 (C), 132.28 (C), 135.54 (CH), 137.80 (C), 142.51 (C), 143.05 (CH), 150.09 (C), 166.11 (C), 167.89 (C). HRMS (ESI) (*m*/*z*): [M + H]^+^ calcd for C_19_H_14_ClN_5_O_2_S: 412.0629, found, 412.0659.

##### 4.1.3.10 *N*′-[4-(1*H*-1,2,4-Triazol-1-yl)benzylidene]-2-[(5-chlorobenzoxazol-2-yl)thio]acetohydrazide (**3j**)

Yield: 80%, M.p.: 256–257 °C. IR ν_max_ (cm^–1^): 3207.62, 3124.68, 3088.03, 2951.09, 2829.57, 1670.35, 1614.42, 1523.76, 1492.90, 1452.40, 1402.25, 1373.32, 1344.38, 1319.31, 1280.73, 1246.02, 1219.01, 1201.65, 1143.79, 1107.14, 1051.20, 979.84, 960.55, 916.19, 889.18, 833.25, 819.75, 790.81, 702.09, 671.23. ^1^H NMR (400 MHz, DMSO-*d*_6_) δ (ppm): 4.29 and 4.72 (2s, 2H), 7.34 (dd, *J* = 8.6 Hz, 2.2 Hz, 1H), 7.67 (dd, *J* = 8.6 Hz, 5.0 Hz, 1H), 7.72–7.74 (m, 1H), 7.87–7.96 (m, 4H), 8.09 and 8.26 (2s, 1H), 8.27 (s, 1H), 9.37 (s, 1H), 11.87 (brs, 1H). ^13^C NMR (100 MHz, DMSO-*d*_6_) δ (ppm): 34.83 (CH_2_), 111.43 (CH), 118.03 (CH), 119.49 (2CH), 124.16 (CH), 128.30 (2CH), 128.51 (C), 133.19 (C), 137.51 (C), 142.45 (CH), 142.51 (C), 142.95 (CH), 150.09 (C), 152.57 (CH), 166.10 (C), 167.91 (C). HRMS (ESI) (*m*/*z*): [M + H]^+^ calcd for C_18_H_13_ClN_6_O_2_S: 413.0582, found, 413.0607.

### 4.2 Biochemistry

#### 4.2.1 Cell Culture and Drug Treatment

A549 and L929 cell lines were purchased from American Type Culture Collection (Manassas, VA, USA). Cells were cultured, and drug treatments were performed as previously explained.^46^

#### 4.2.2 MTT Test

MTT assay was conducted as previously described^47^ with slight modifications.^48^ Cisplatin was used as a positive control. The assay was performed in triplicate. Half-maximal inhibitory concentration (IC_50_) data (μM) were expressed as mean ± SD.

#### 4.2.3 Flow Cytometric Analyses of Apoptosis

FITC Annexin V Apoptosis Detection Kit (BD Pharmingen, San Jose, CA, USA) was applied using a CytoFLEX flow cytometer (Beckman Coulter, Indianapolis, USA) in accordance with the manufacturer’s instructions following the 24 h incubation of A549 cells with compounds **3a**, **3e**, **3g**, **3i**, and cisplatin at 91.35, 176.23, 46.60, 83.59, and 23.89 μM, respectively.

#### 4.2.4 Determination of Akt Inhibition

Akt Colorimetric In-Cell ELISA Kit (Thermo Fisher Scientific, Waltham, MA, USA) was used in accordance with the instructions provided by the manufacturer after A549 cells were incubated with compounds **3a** (22.84, 45.68, and 91.35 μM), **3e** (44.06, 88.12, and 176.23 μM), **3g** (11.65, 23.30, and 46.60 μM), **3i** (20.90, 41.80, and 83.59 μM), cisplatin (5.97, 11.95, and 23.89 μM), and GSK690693 (3.61, 7.23, and 14.45 μM) for 24 h. The assay was performed in triplicate. IC_50_ data (μM) were expressed as mean ± SD.

#### 4.2.5 Statistical Analyses

Statistical Package for the Social Sciences (SPSS) for Windows 15.0 was used for statistical analysis. Comparisons were performed by one-way ANOVA test for normally distributed continuous variables, and post hoc analyses of group differences were expressed by the Tukey test.

### 4.3 *In Silico* Studies

The crystal structure of human Akt2 in complex with GSK690693 was downloaded from Protein Data Bank (PDB ID: 3D0E at 2.00 Å resolution). This raw protein structure was initially prepared^49^ by adding missing hydrogens, correcting bond orders, and removing all heteroatoms except the native ligand. The structure was then optimized to address any overlapping hydrogens, and finally, restrained minimization was carried out.

The binding site of Akt2 was defined by computing grid file using GSK690693 as a reference agent. The size of enclosing box that represents grid was extended to allow docking of the ligands with length <20 Å. Glide XP module^50^ was selected for molecular docking. LigPrep module^51^ was used to prepare ligands for *in silico* calculations to consider all possible geometries, tautomers, and also protonation states at physiological conditions. Important molecular descriptors related to ADME were predicted using QikProp module.^52^

## Supporting Information Available

IR, ^1^H NMR, ^13^C NMR, and HRMS spectra of compounds **1**, **2**, and **3a–j** (PDF)
