# Supplementary material for: Design, Synthesis, and Evaluation of a New Series of Hydrazones as Small-Molecule Akt Inhibitors for NSCLC Therapy
Source: ACS Omega. 2023 May 24;8(22):20056–65. doi: 10.1021/acsomega.3c02331 (PMC10249096; doi:10.1021/acsomega.3c02331)
Supplement: Supplementary file 1 — ao3c02331_si_001.pdf [file ao3c02331_si_001.pdf]

Supporting Information

Design, Synthesis and Evaluation of A New Series of Hydrazones as Small Molecule Akt Inhibitors for NSCLC Therapy

Burak Erdönmez[1], Mehlika Dilek Altıntop[2,*], Gülşen Akalın Çiftçi[3], Ahmet Özdemir[2], Abdulilah Ece[4]

[1] *Department of Pharmaceutical Chemistry, Graduate School of Health Sciences, Anadolu University, 26470 Eskişehir, Turkey*

[2] *Department of Pharmaceutical Chemistry, Faculty of Pharmacy, Anadolu University, 26470 Eskişehir, Turkey*

[3] *Department of Biochemistry, Faculty of Pharmacy, Anadolu University, 26470 Eskişehir, Turkey*

[4] *Department of Pharmaceutical Chemistry, Faculty of Pharmacy, Biruni University, 34010 Istanbul, Turkey*

List of Contents

Figure	Page
**Figure S1.** IR spectrum of compound **1**	**4**
**Figure S2.** ^{1}H NMR spectrum of compound **1**	**5**
**Figure S3.** ^{13}C NMR spectrum of compound **1**	**5**
**Figure S4.** HRMS spectrum of compound **1**	**6**
**Figure S5.** IR spectrum of compound **2**	**7**
**Figure S6.** ^{1}H NMR spectrum of compound **2**	**8**
**Figure S7.** ^{13}C NMR spectrum of compound **2**	**8**
**Figure S8.** HRMS spectrum of compound **2**	**9**
**Figure S9.** IR spectrum of compound **3a**	**10**
**Figure S10.** ^{1}H NMR spectrum of compound **3a**	**11**
**Figure S11.** ^{13}C NMR spectrum of compound **3a**	**11**
**Figure S12.** HRMS spectrum of compound **3a**	**12**
**Figure S13.** IR spectrum of compound **3b**	**13**
**Figure S14.** ^{1}H NMR spectrum of compound **3b**	**14**
**Figure S15.** ^{13}C NMR spectrum of compound **3b**	**14**
**Figure S16.** HRMS spectrum of compound **3b**	**15**
**Figure S17.** IR spectrum of compound **3c**	**16**
**Figure S18.** ^{1}H NMR spectrum of compound **3c**	**17**
**Figure S19.** ^{13}C NMR spectrum of compound **3c**	**17**
**Figure S20.** HRMS spectrum of compound **3c**	**18**
**Figure S21.** IR spectrum of compound **3d**	**19**
**Figure S22.** ^{1}H NMR spectrum of compound **3d**	**20**
**Figure S23.** ^{13}C NMR spectrum of compound **3d**	**20**
**Figure S24.** HRMS spectrum of compound **3d**	**21**
**Figure S25.** IR spectrum of compound **3e**	**22**
**Figure S26.** ^{1}H NMR spectrum of compound **3e**	**23**
**Figure S27.** ^{13}C NMR spectrum of compound **3e**	**23**

Figure S28. HRMS spectrum of compound **3e** **24**

Figure S29. IR spectrum of compound **3f** **25**

Figure S30. ^{1}H NMR spectrum of compound **3f** **26**

Figure S31. ^{13}C NMR spectrum of compound **3f** **26**

Figure S32. HRMS spectrum of compound **3f** **27**

Figure S33. IR spectrum of compound **3g** **28**

Figure S34. ^{1}H NMR spectrum of compound **3g** **29**

Figure S35. ^{13}C NMR spectrum of compound **3g** **29**

Figure S36. HRMS spectrum of compound **3g** **30**

Figure S37. IR spectrum of compound **3h** **31**

Figure S38. ^{1}H NMR spectrum of compound **3h** **32**

Figure S39. ^{13}C NMR spectrum of compound **3h** **32**

Figure S40. HRMS spectrum of compound **3h** **33**

Figure S41. IR spectrum of compound **3i** **34**

Figure S42. ^{1}H NMR spectrum of compound **3i** **35**

Figure S43. ^{13}C NMR spectrum of compound **3i** **35**

Figure S44. HRMS spectrum of compound **3i** **36**

Figure S45. IR spectrum of compound **3j** **37**

Figure S46. ^{1}H NMR spectrum of compound **3j** **38**

Figure S47. ^{13}C NMR spectrum of compound **3j** **38**

Figure S48. HRMS spectrum of compound **3j** **39**

Figure S1. IR spectrum of compound **1**

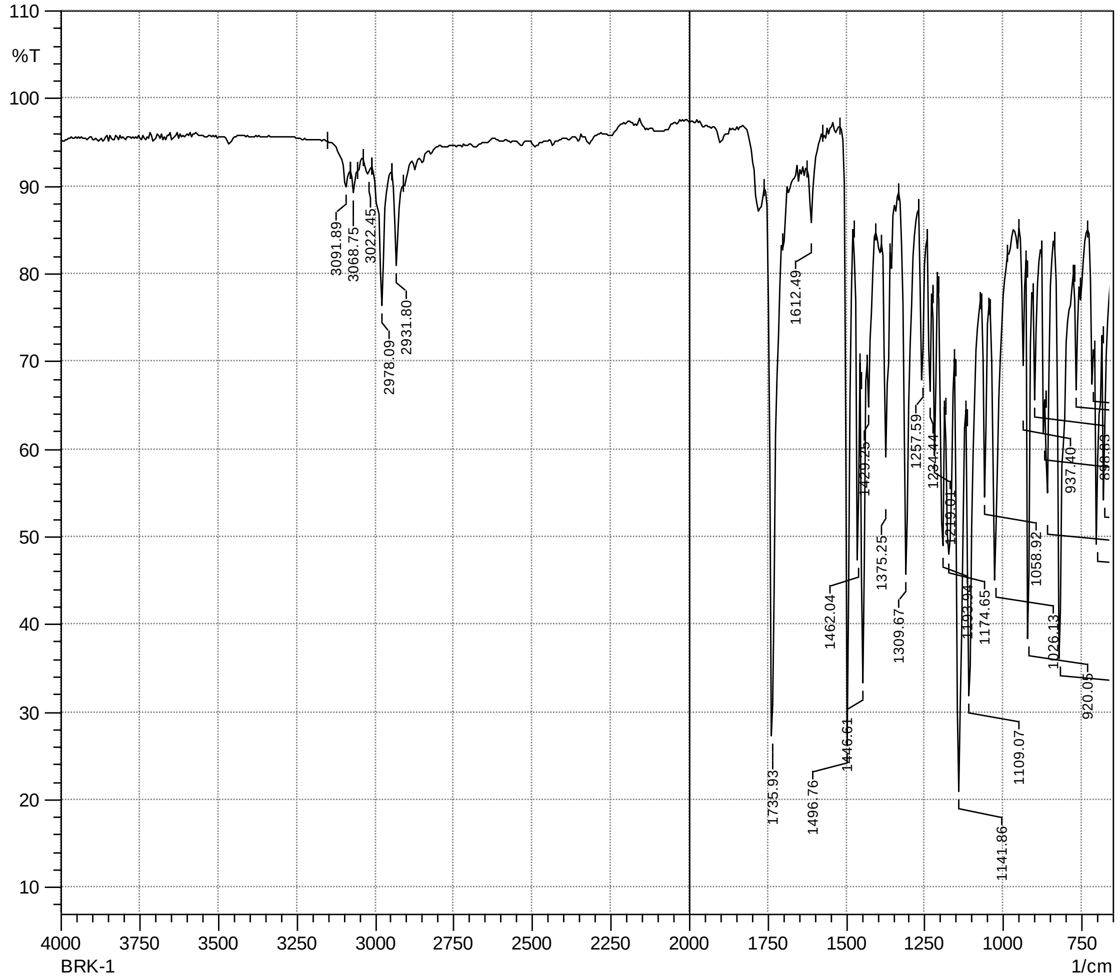

Figure S2. ^{1}H NMR spectrum of compound **1**

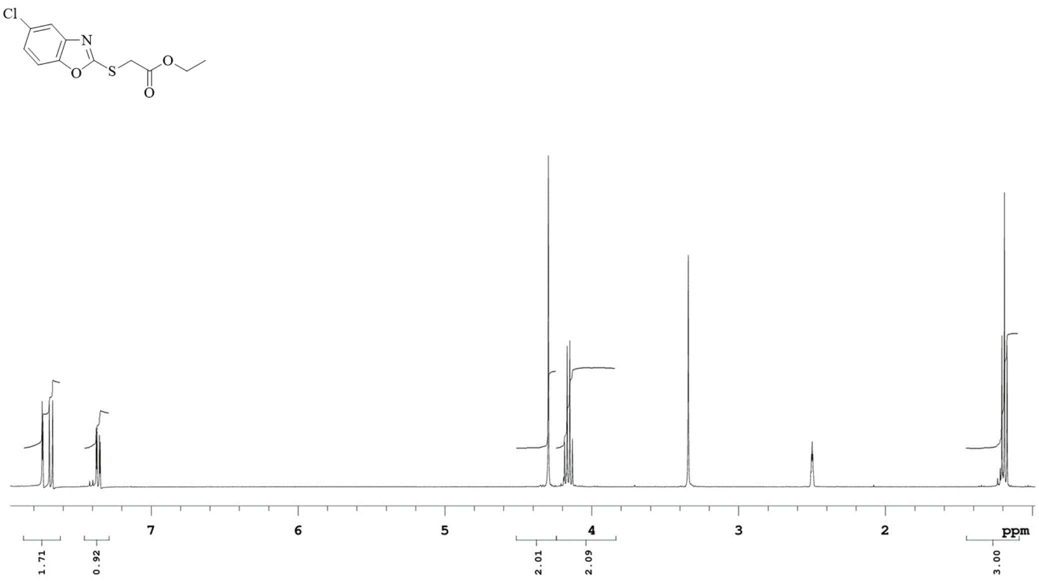

Figure S3. ^{13}C NMR spectrum of compound **1**

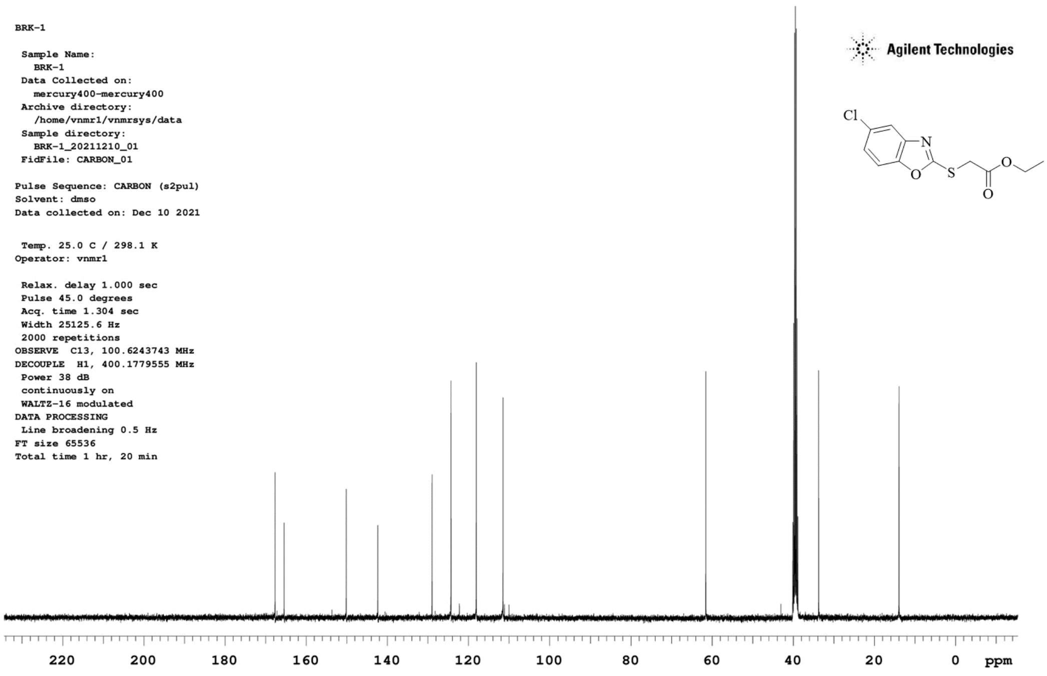

Figure S4. HRMS spectrum of compound **1**

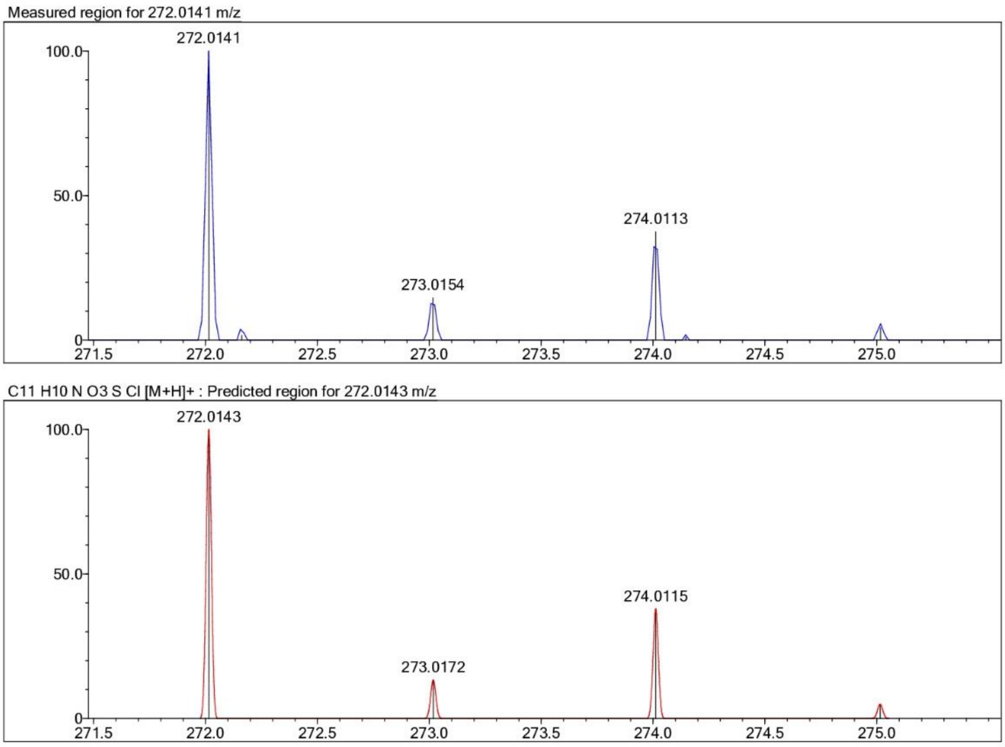

Rank	Score	Formula (M)	Ion	Meas. m/z	Pred. m/z	Df. (mDa)	Df. (ppm)	Iso	DBE
1	90.75	C11 H10 N O3 S Cl	[M+H]+	272.0141	272.0143	-0.2	-0.74	90.75	7.0

Figure S5. IR spectrum of compound **2**

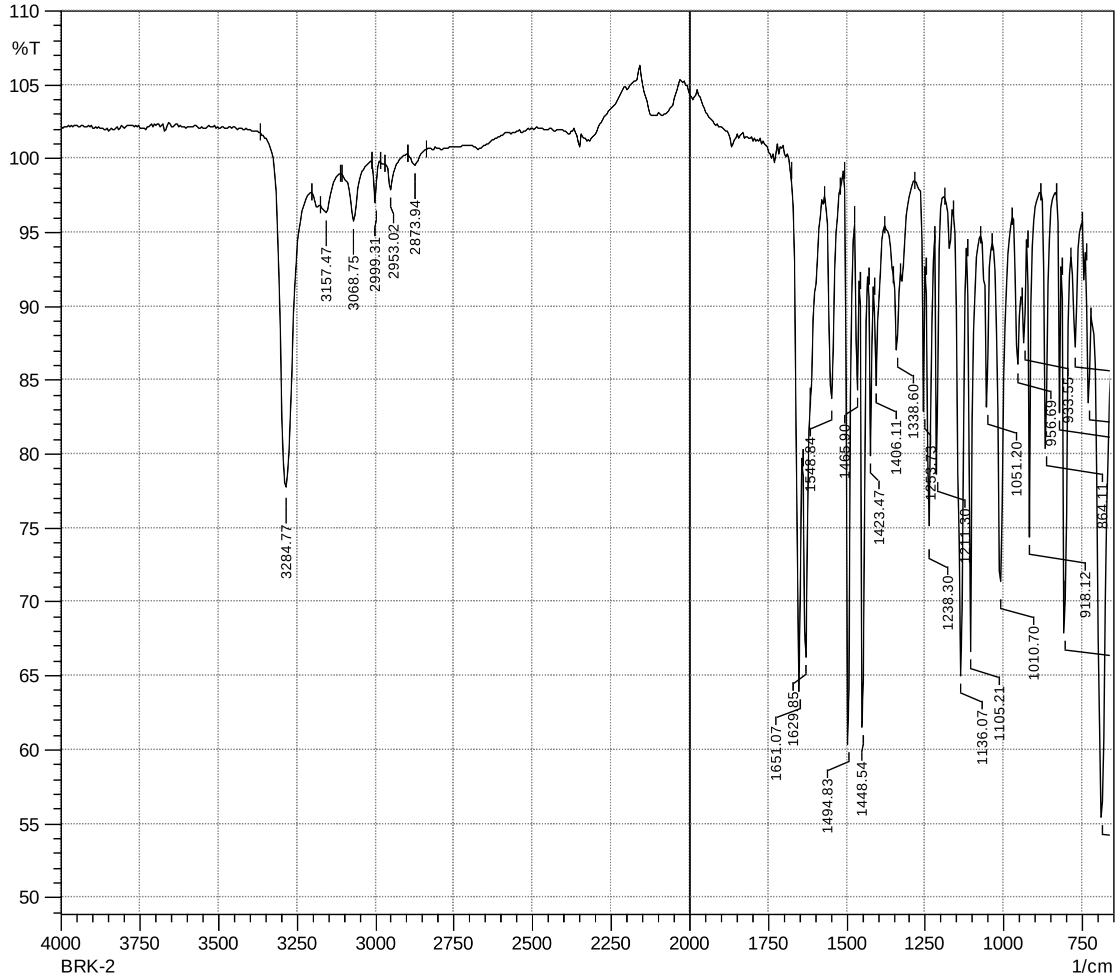

Figure S6. ^{1}H NMR spectrum of compound **2**

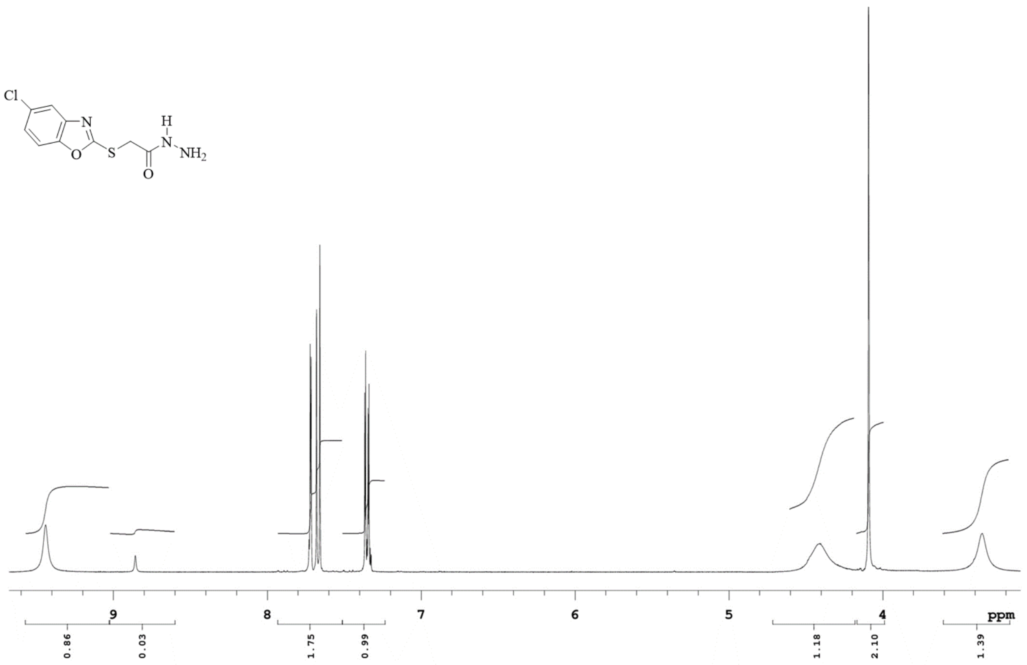

Figure S7. ^{13}C NMR spectrum of compound **2**

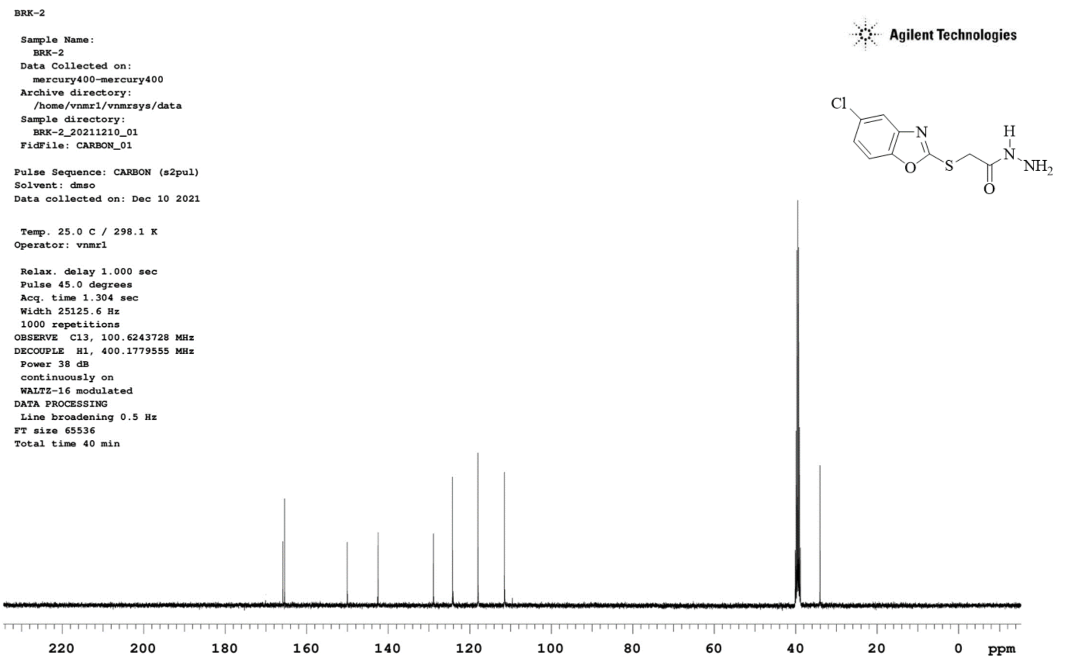

Figure S8. HRMS spectrum of compound **2**

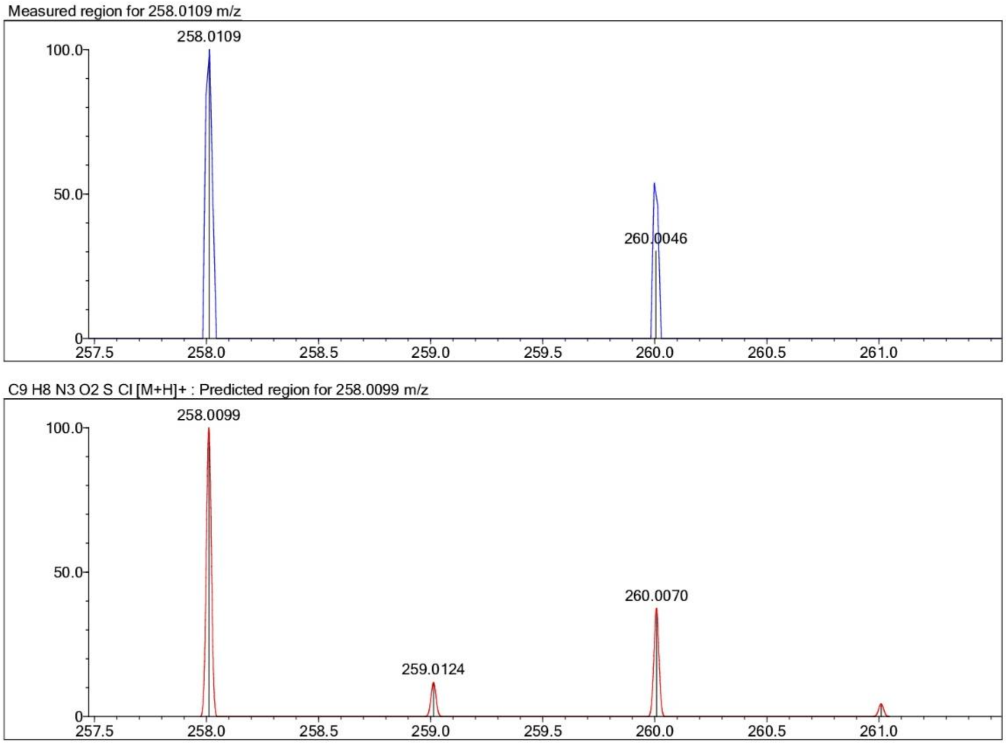

Rank	Score	Formula (M)	Ion	Meas. m/z	Pred. m/z	Df. (mDa)	Df. (ppm)	Iso	DBE
1	45.60	C9 H8 N3 O2 S Cl	[M+H]+	258.0109	258.0099	1.0	3.88	49.14	7.0

Figure S9. IR spectrum of compound **3a**

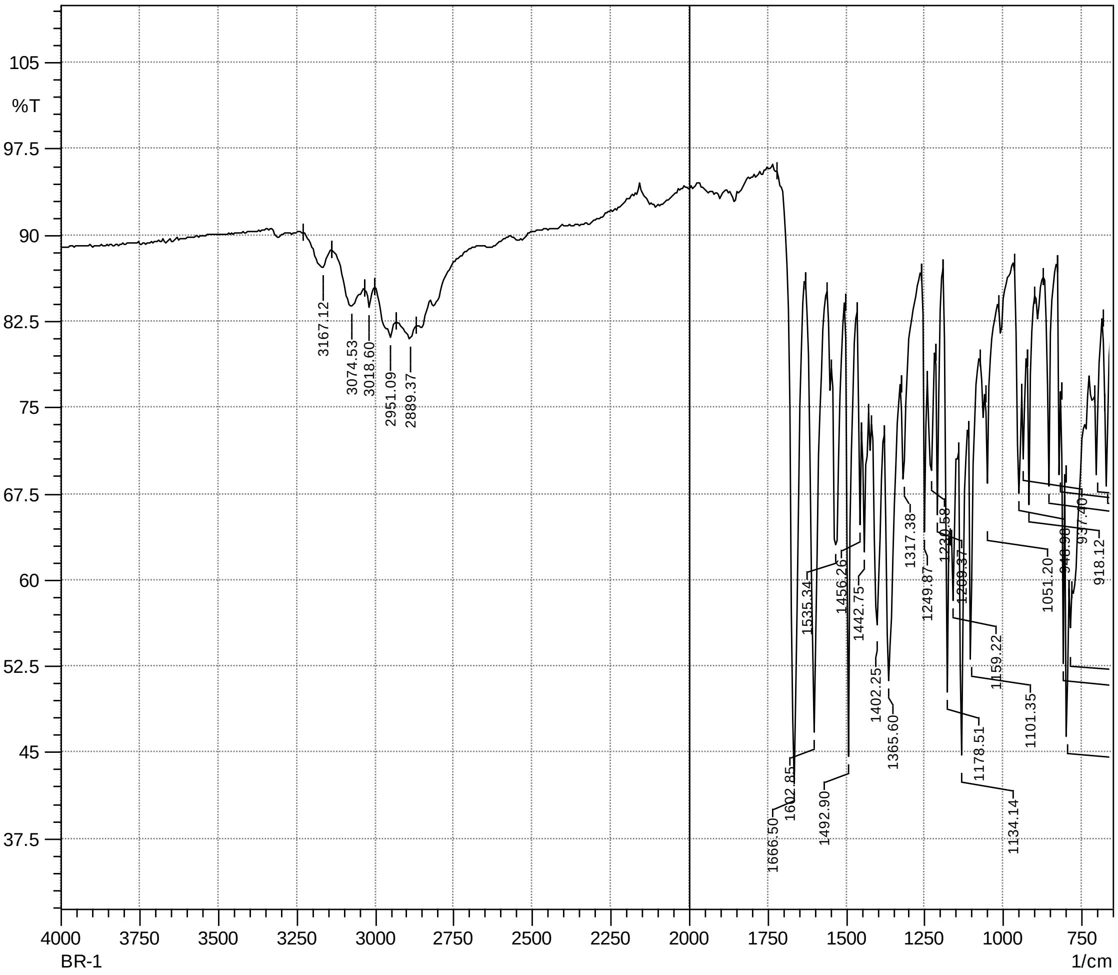

Figure S10. ^{1}H NMR spectrum of compound **3a**

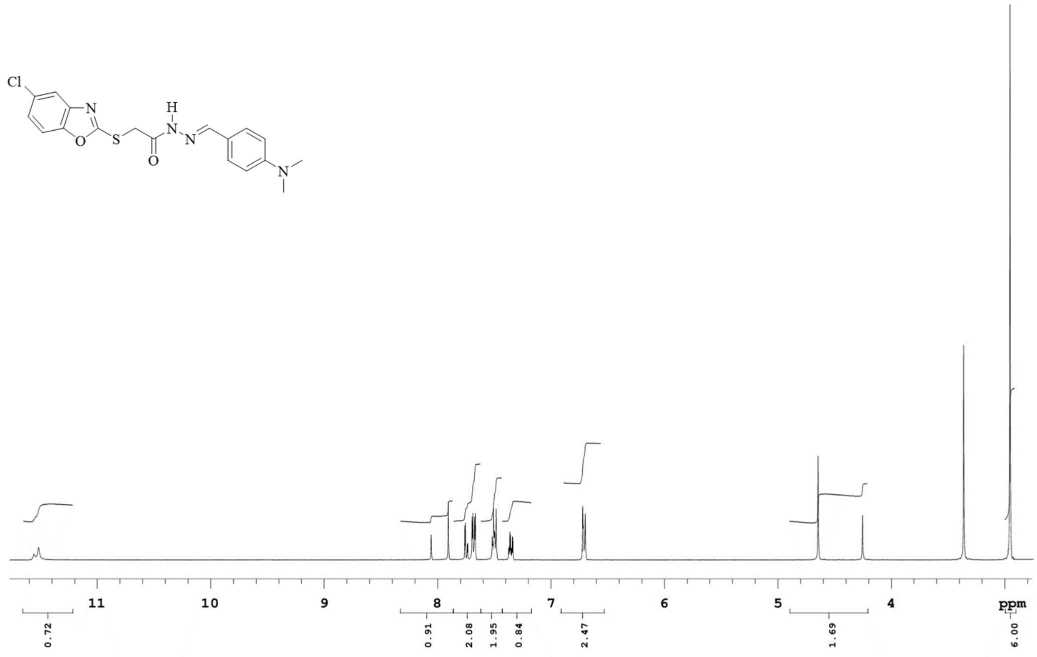

Figure S11. ^{13}C NMR spectrum of compound **3a**

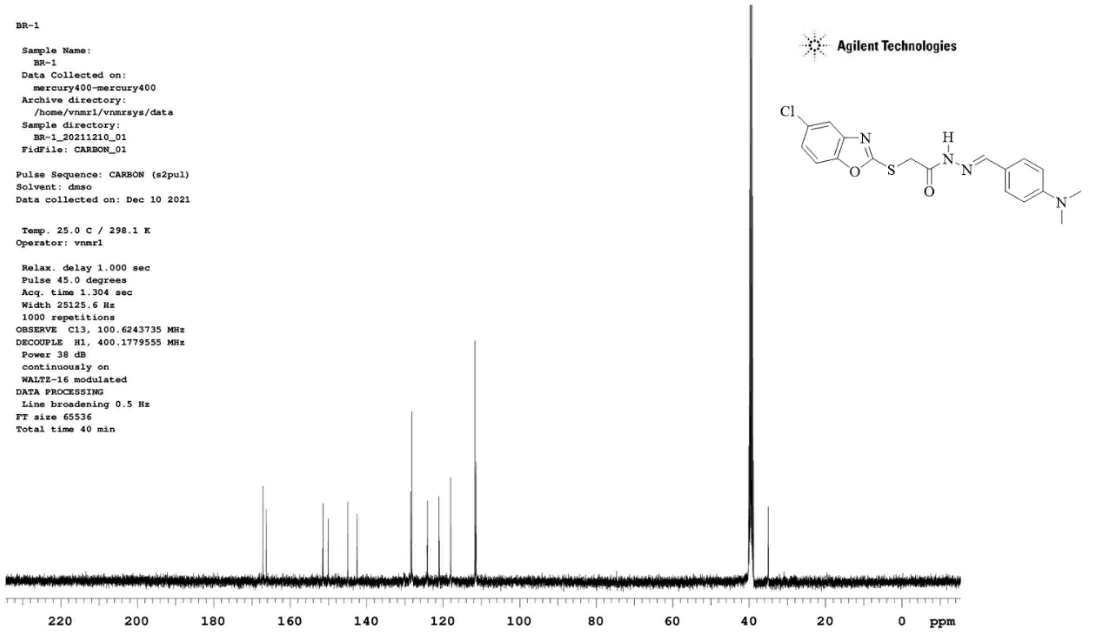

Figure S12. HRMS spectrum of compound **3a**

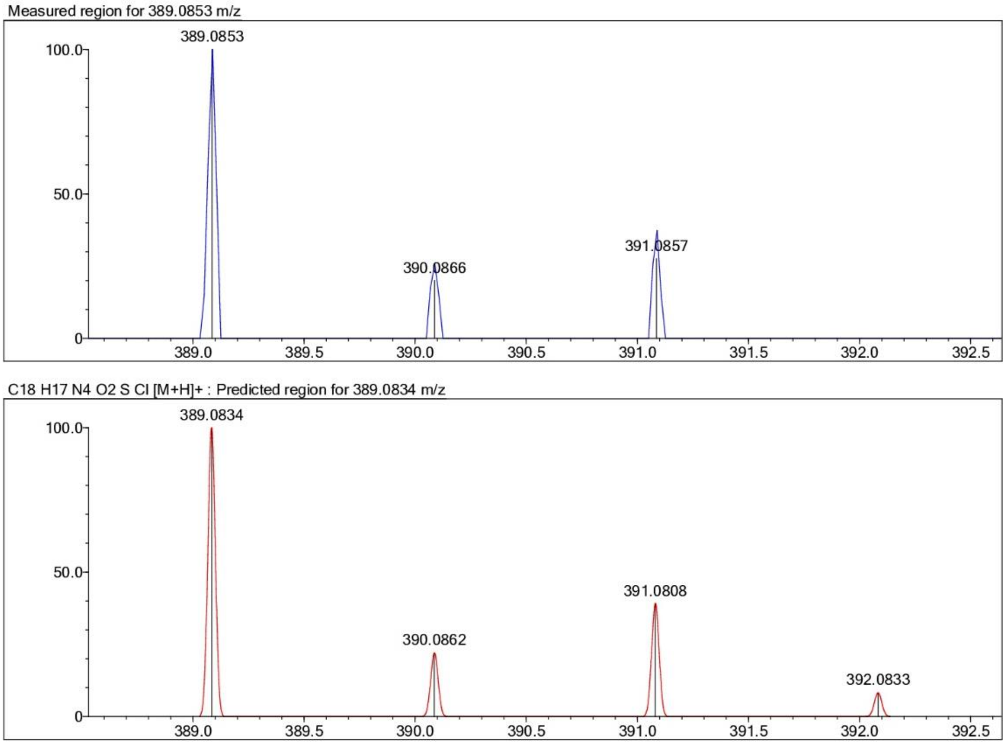

Rank	Score	Formula (M)	Ion	Meas. m/z	Pred. m/z	Df. (mDa)	Df. (ppm)	Iso	DBE
1	52.83	C18 H17 N4 O2 S Cl	[M+H]+	389.0853	389.0834	1.9	4.88	58.51	12.0

Figure S13. IR spectrum of compound **3b**

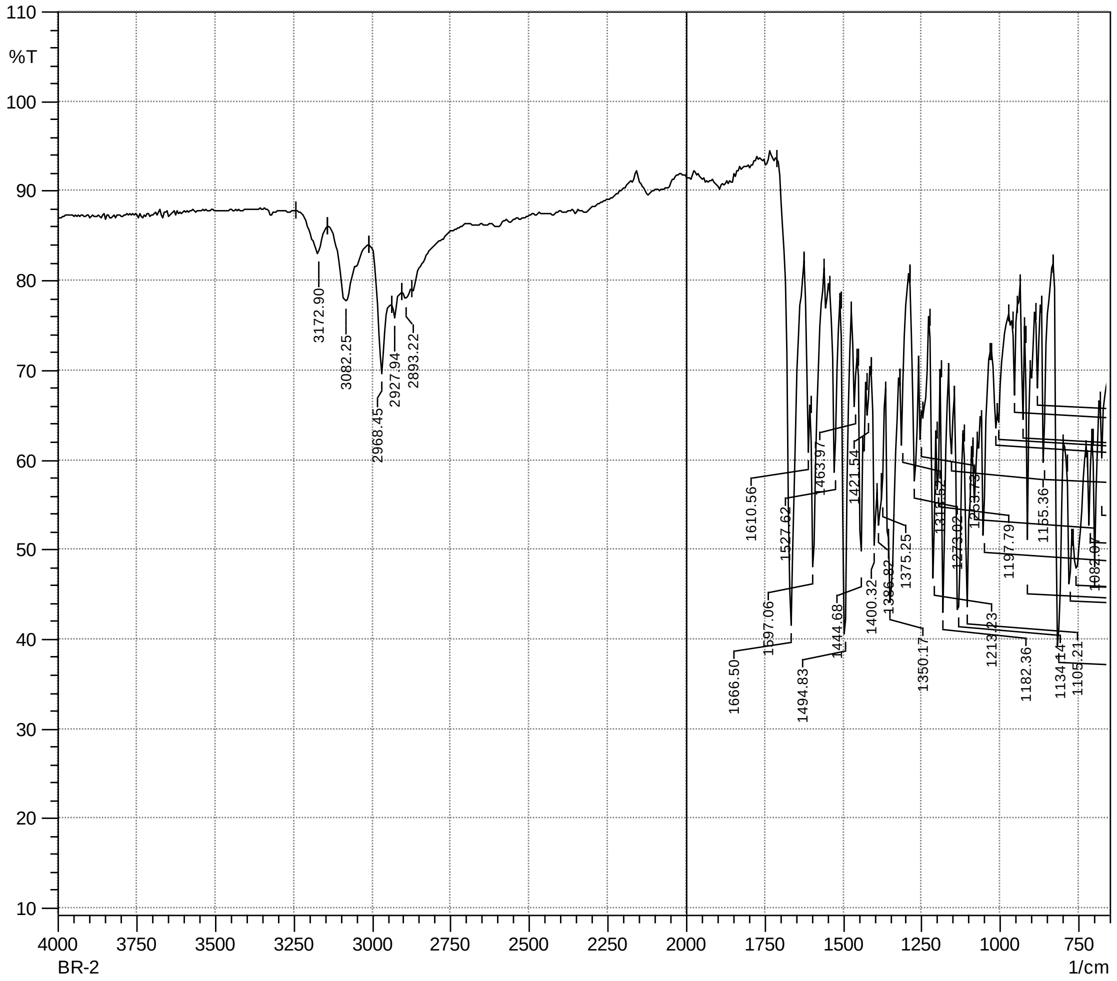

Figure S14. ^{1}H NMR spectrum of compound **3b**

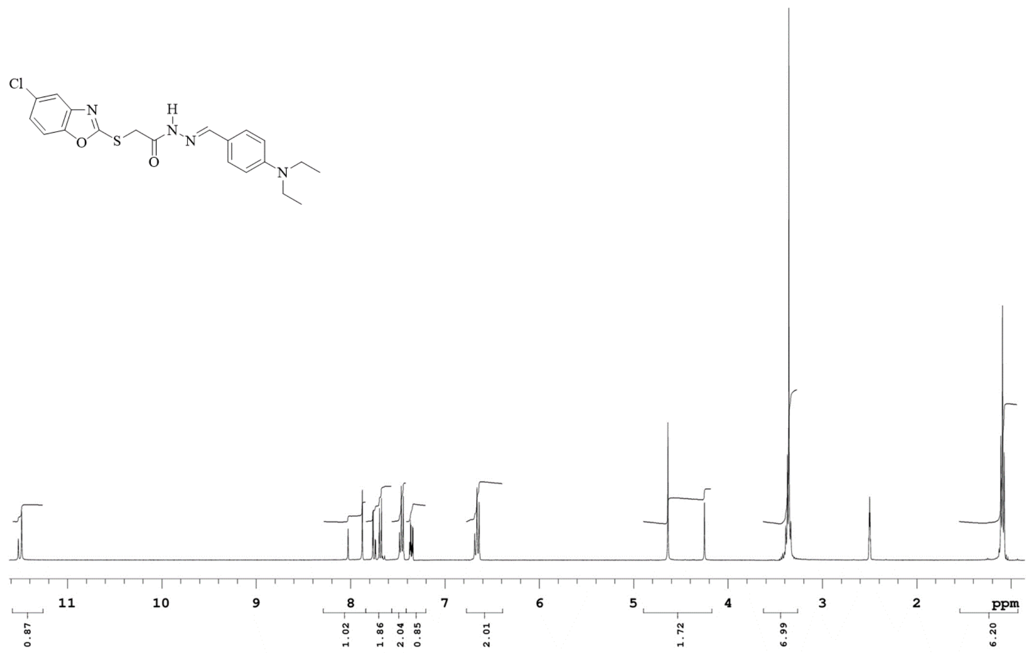

Figure S15. ^{13}C NMR spectrum of compound **3b**

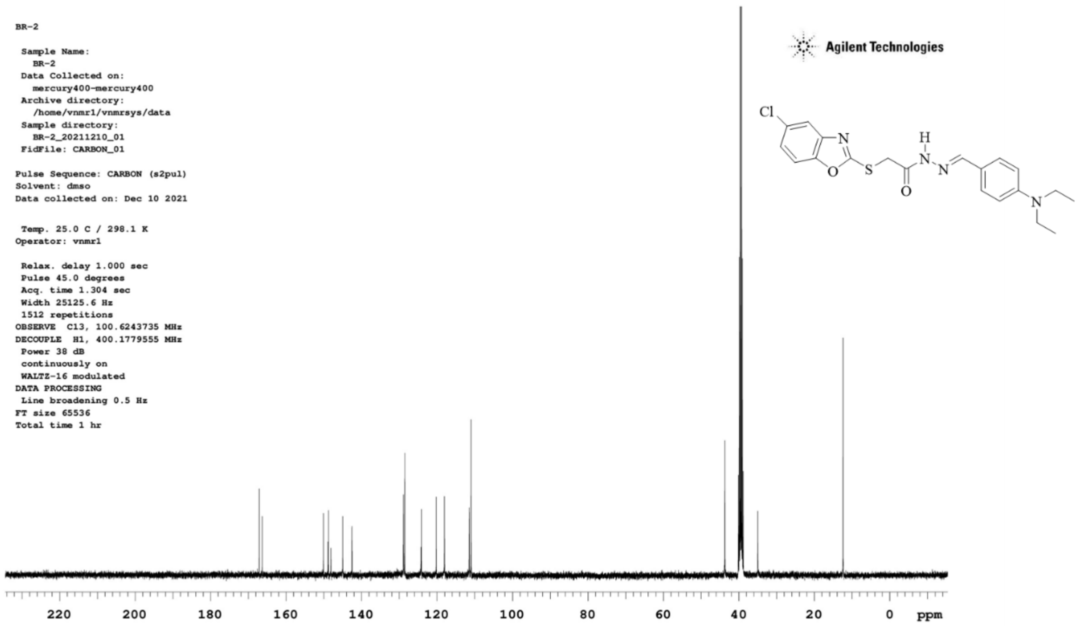

Figure S16. HRMS spectrum of compound **3b**

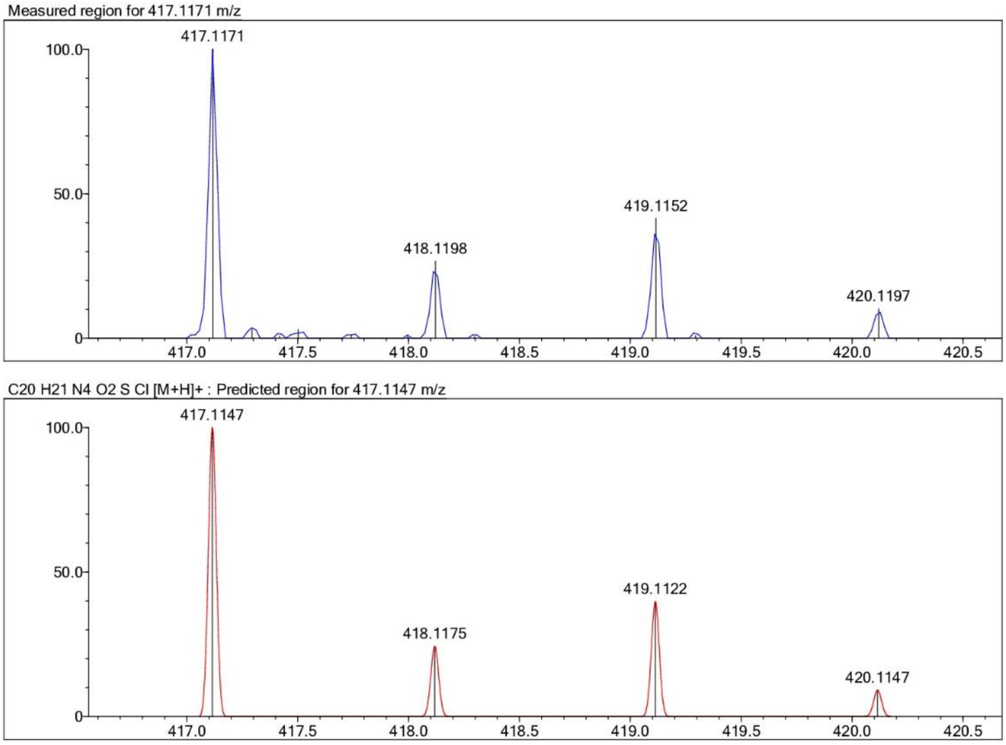

Rank	Score	Formula (M)	Ion	Meas. m/z	Pred. m/z	Df. (mDa)	Df. (ppm)	Iso	DBE
1	73.83	C20 H21 N4 O2 S Cl	[M+H]+	417.1171	417.1147	2.4	5.75	89.49	12.0

Figure S17. IR spectrum of compound **3c**

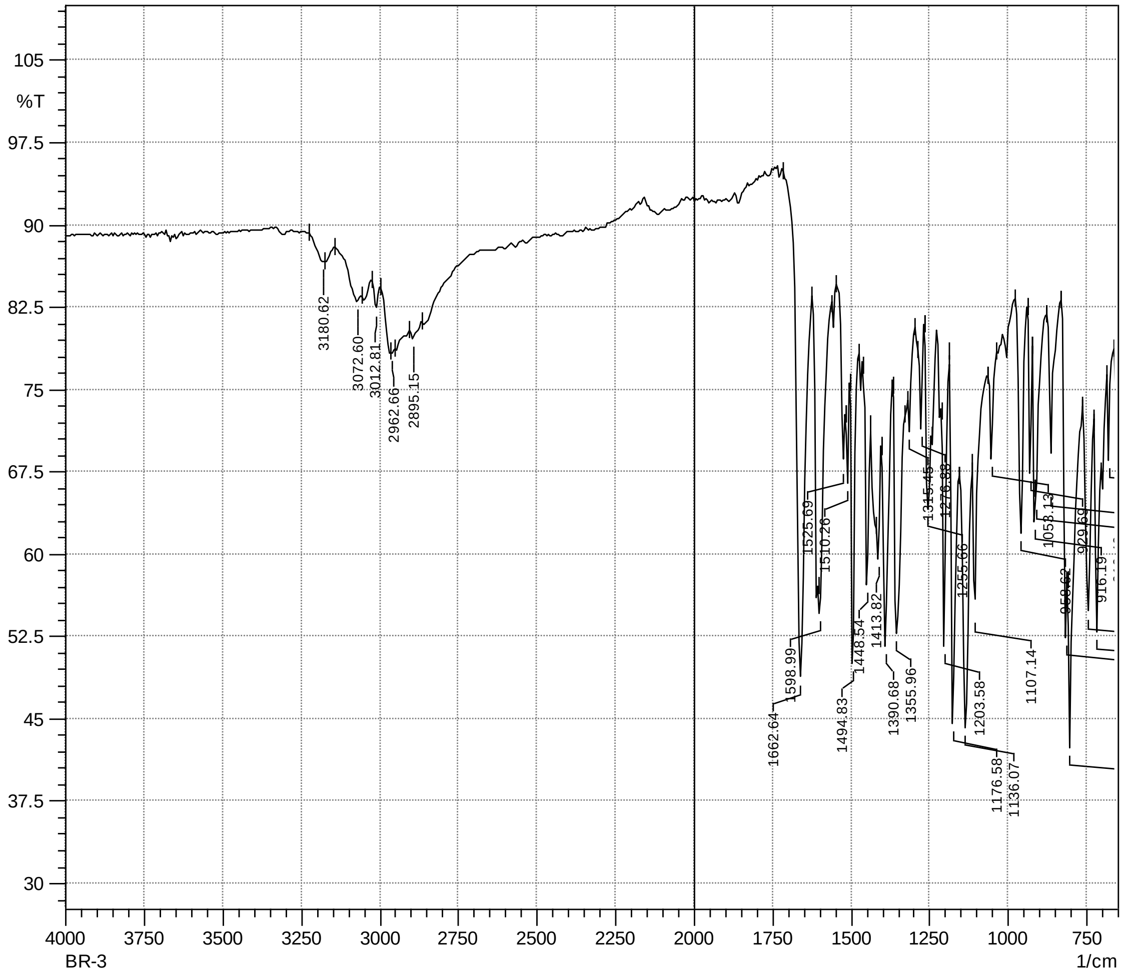

Figure S18. ^{1}H NMR spectrum of compound **3c**

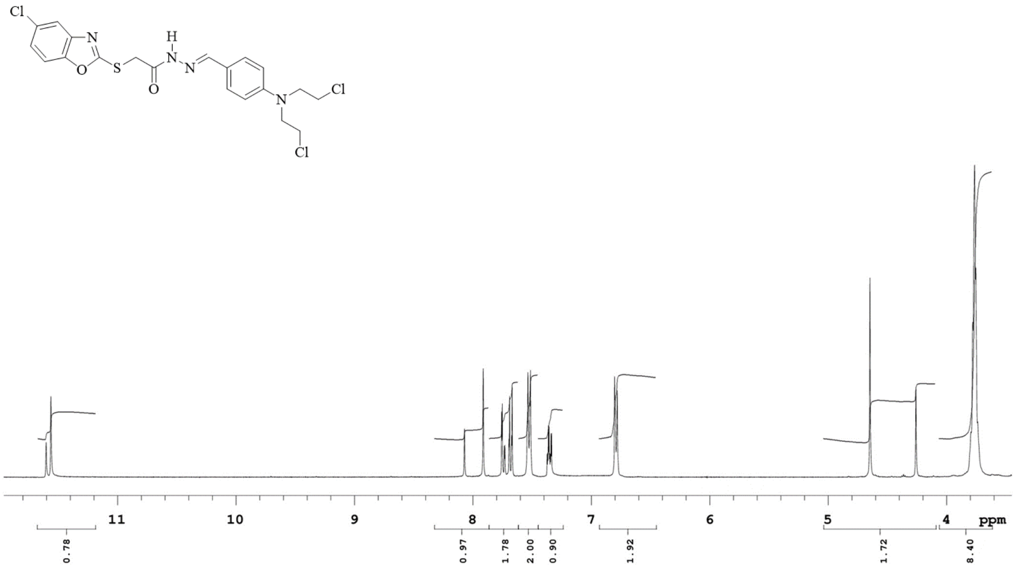

Figure S19. ^{13}C NMR spectrum of compound **3c**

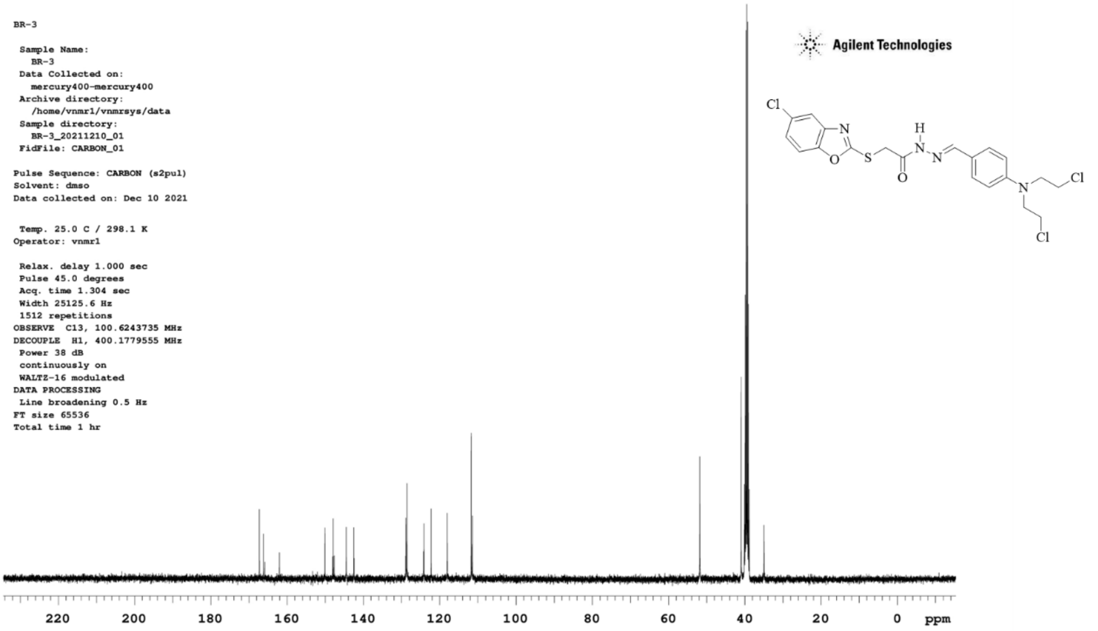

Figure S20. HRMS spectrum of compound **3c**

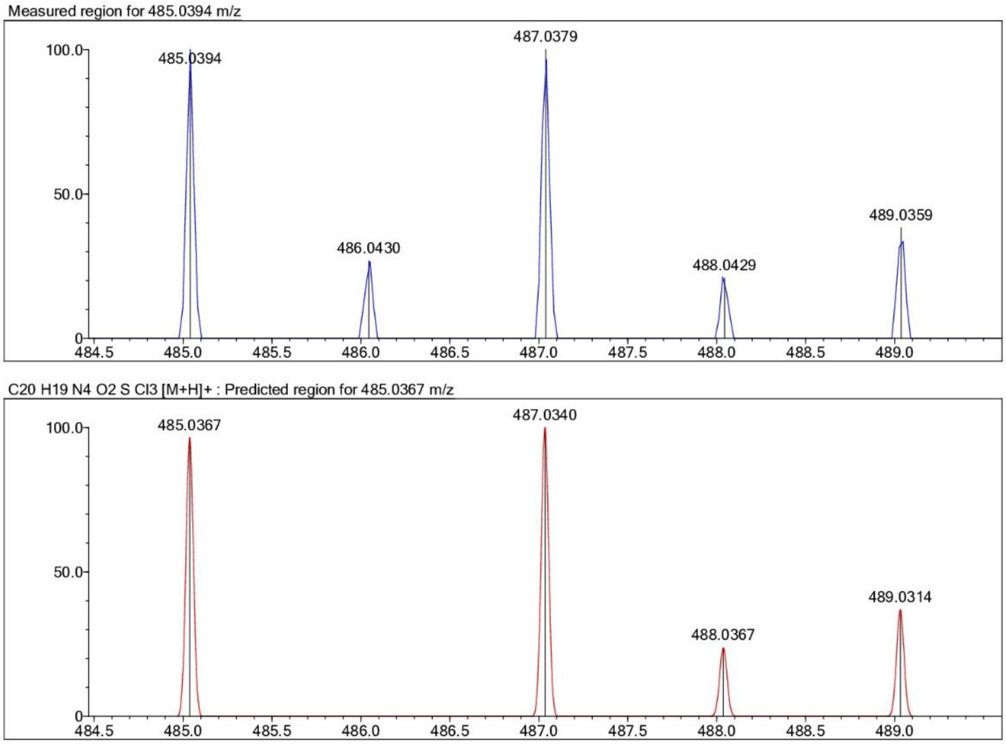

Rank	Score	Formula (M)	Ion	Meas. m/z	Pred. m/z	Df. (mDa)	Df. (ppm)	Iso	DBE
1	50.92	C20 H19 N4 O2 S Cl3	[M+H]+	485.0394	485.0367	2.7	5.57	60.40	12.0

Figure S21. IR spectrum of compound **3d**

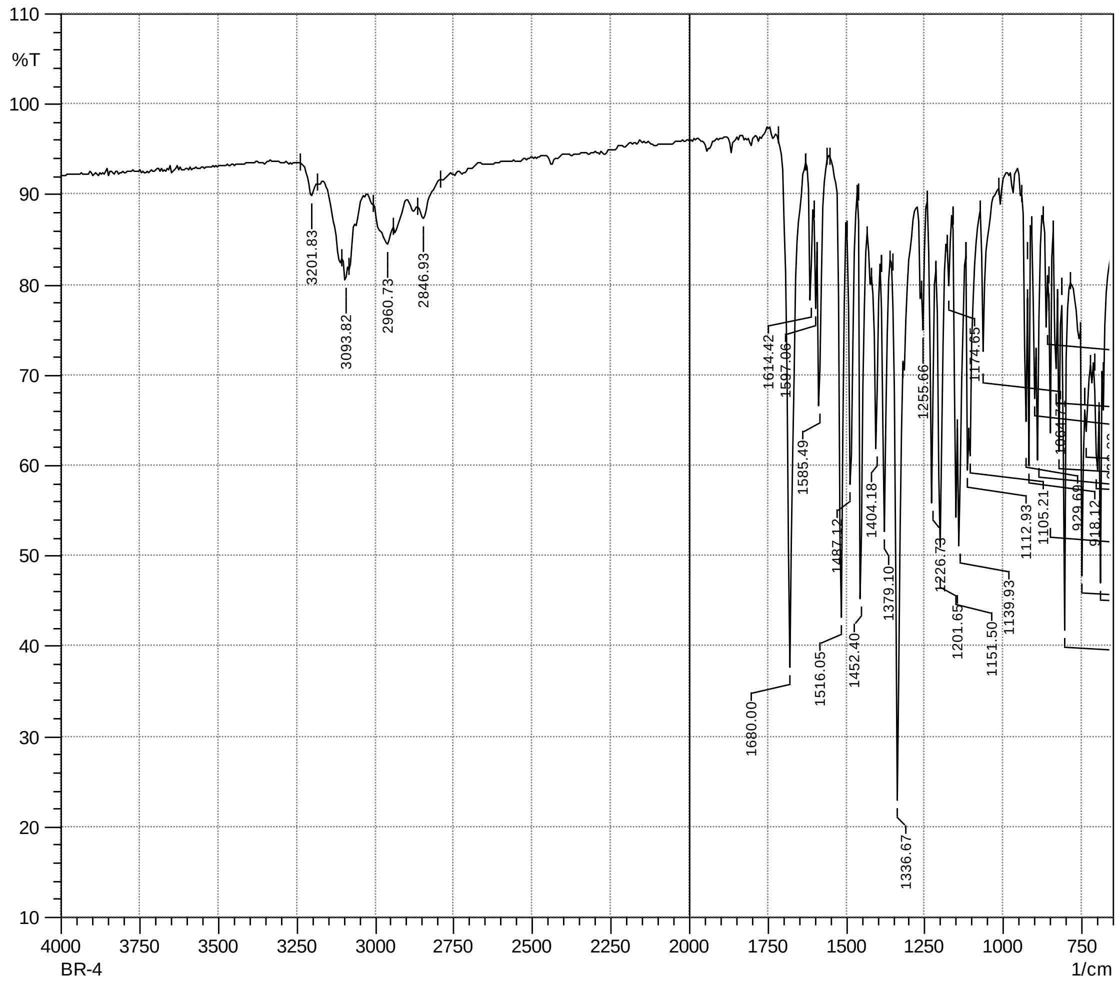

Figure S22. ^{1}H NMR spectrum of compound **3d**

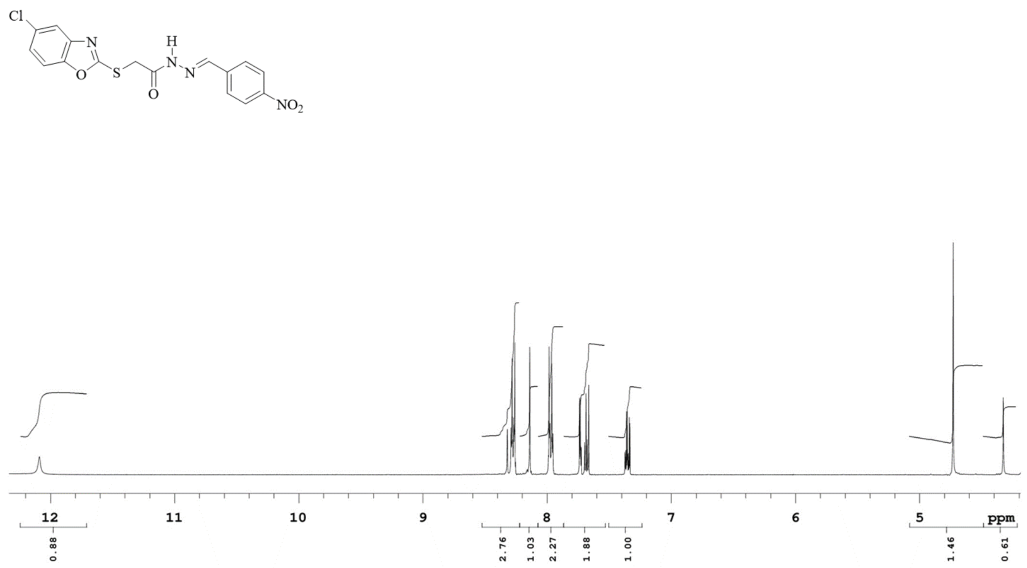

Figure S23. ^{13}C NMR spectrum of compound **3d**

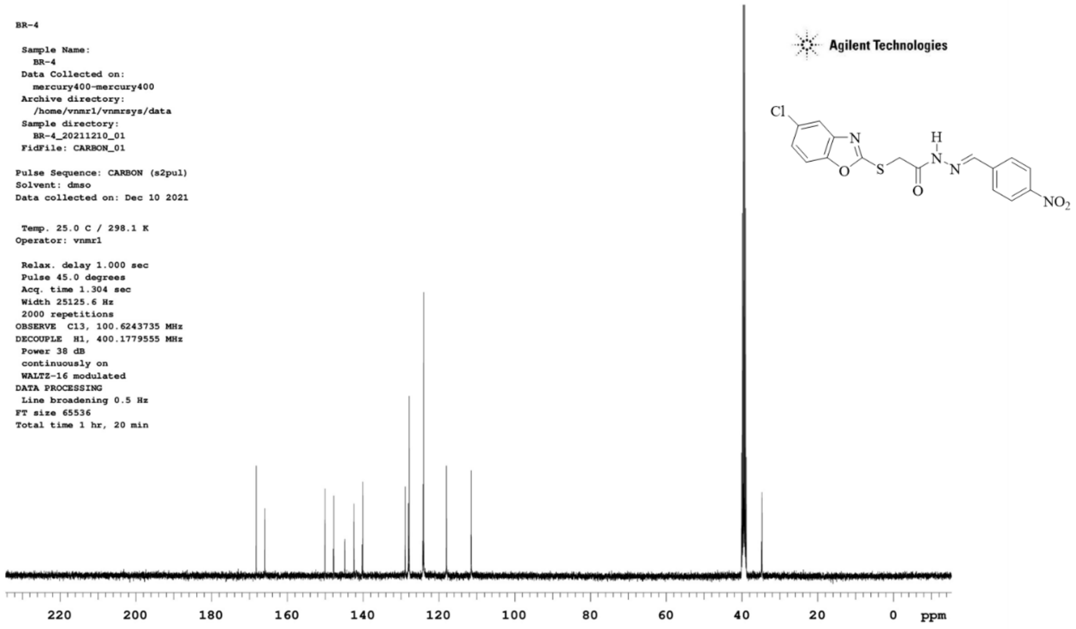

Figure S24. HRMS spectrum of compound **3d**

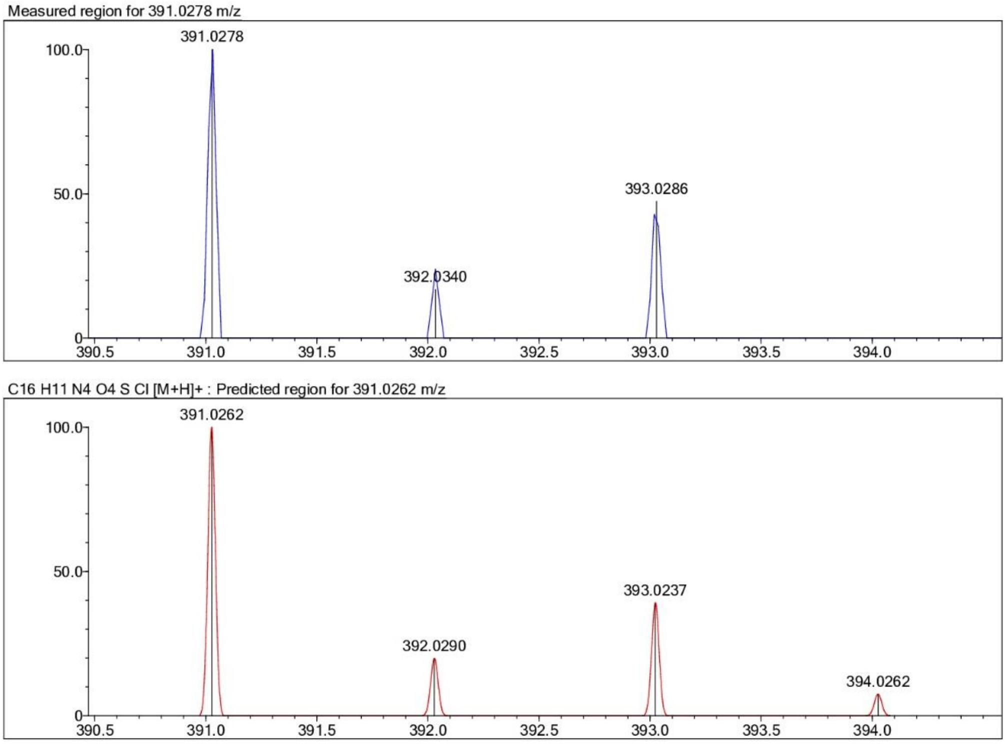

Rank	Score	Formula (M)	Ion	Meas. m/z	Pred. m/z	Df. (mDa)	Df. (ppm)	Iso	DBE
1	57.36	C16 H11 N4 O4 S Cl	[M+H]+	391.0278	391.0262	1.6	4.09	62.16	13.0

Figure S25. IR spectrum of compound **3e**

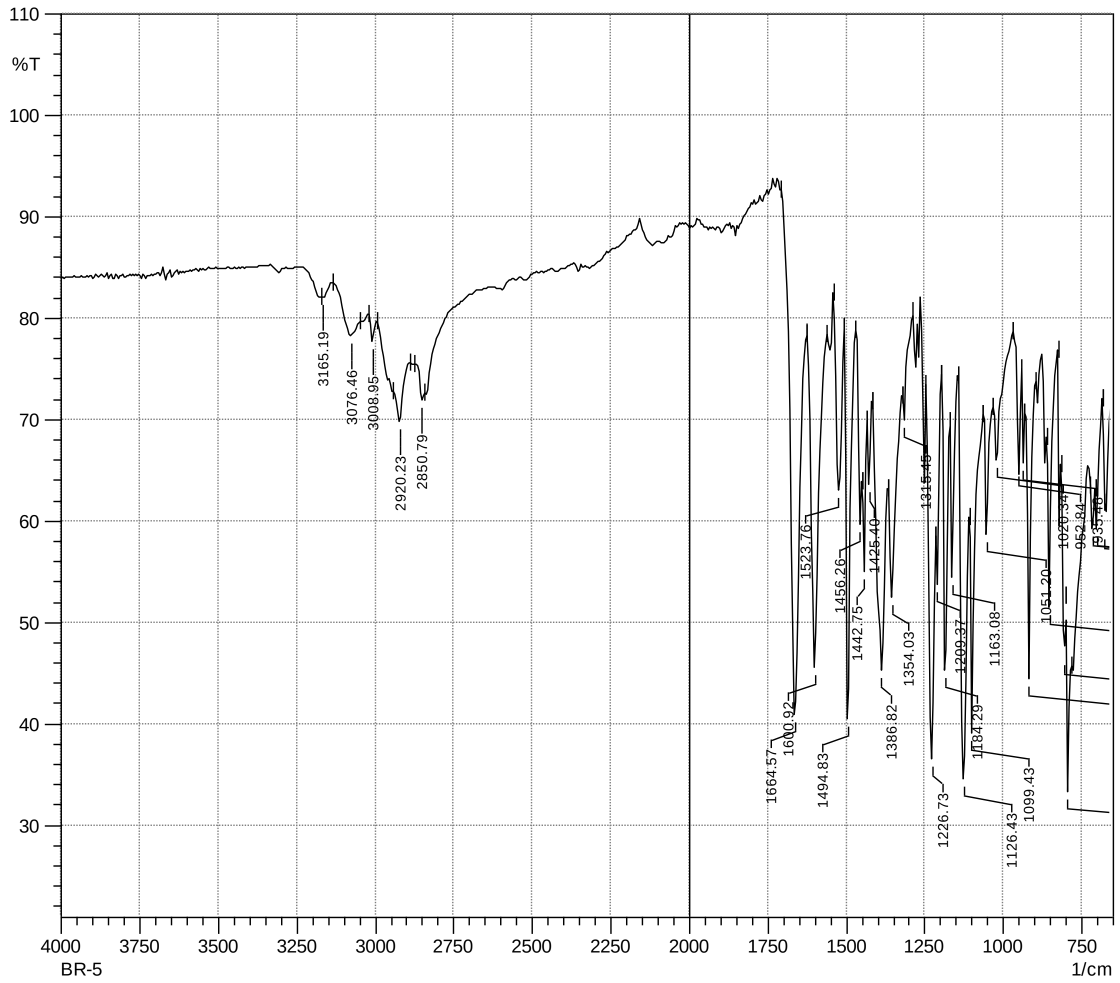

Figure S26. ^{1}H NMR spectrum of compound **3e**

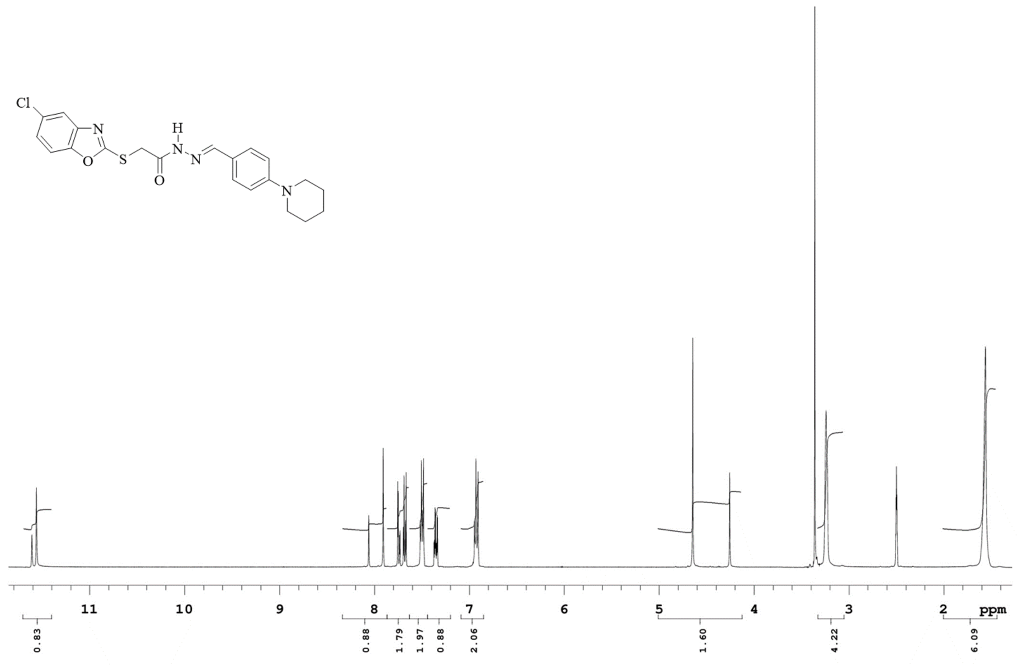

Figure S27. ^{13}C NMR spectrum of compound **3e**

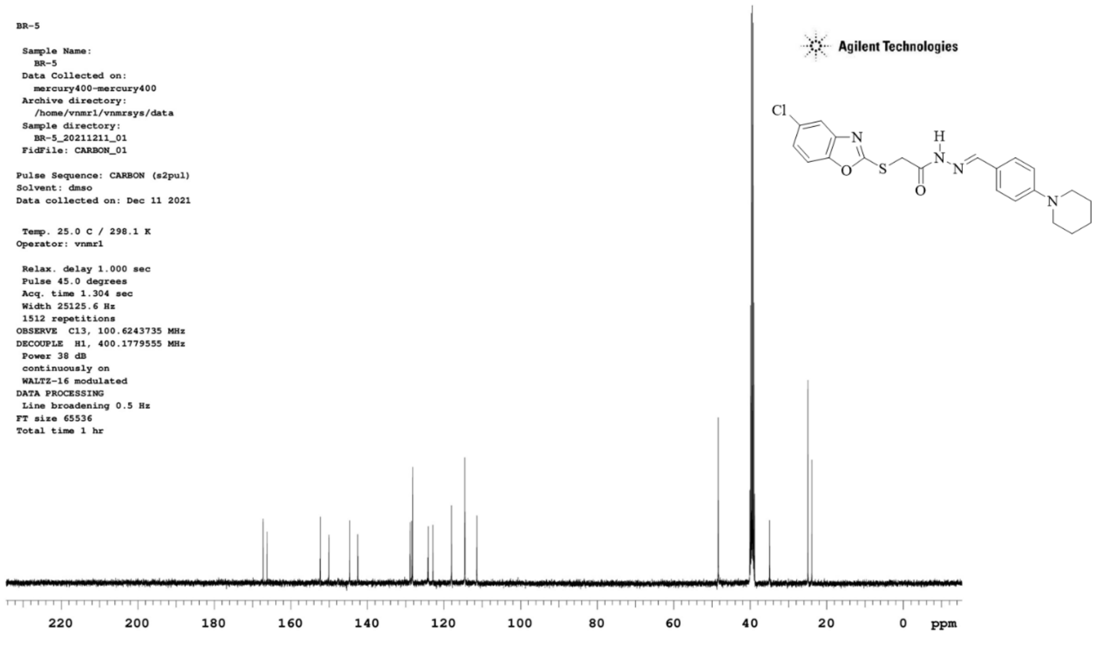

Figure S28. HRMS spectrum of compound **3e**

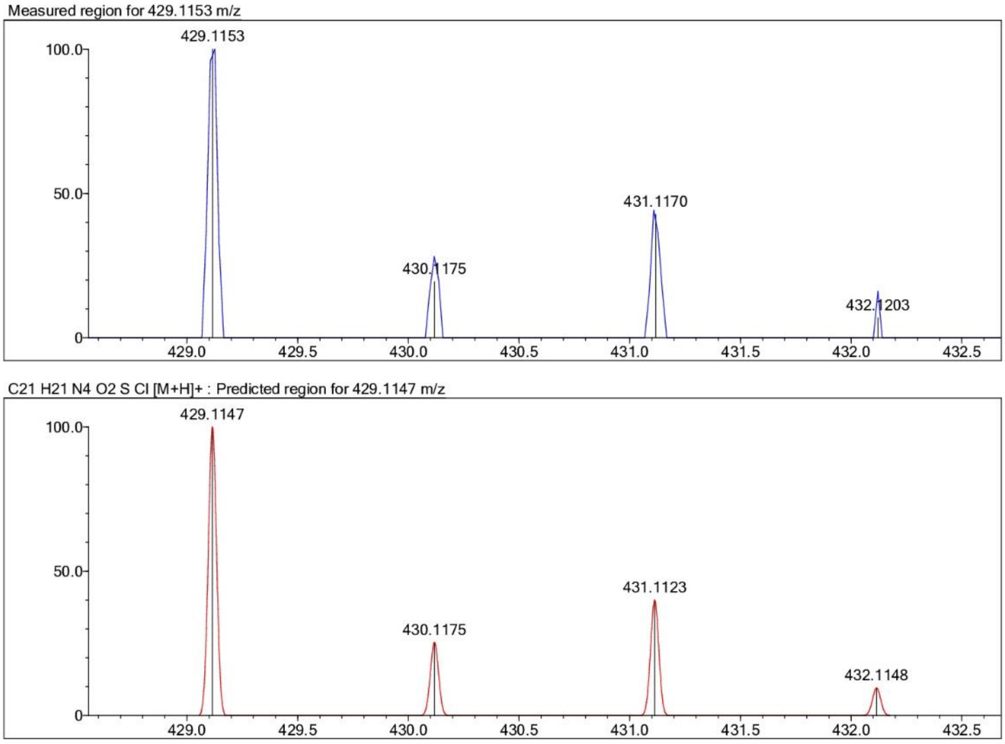

Rank	Score	Formula (M)	Ion	Meas. m/z	Pred. m/z	Df. (mDa)	Df. (ppm)	Iso	DBE
1	83.18	C21 H21 N4 O2 S Cl	[M+H]+	429.1153	429.1147	0.6	1.40	84.02	13.0

Figure S29. IR spectrum of compound **3f**

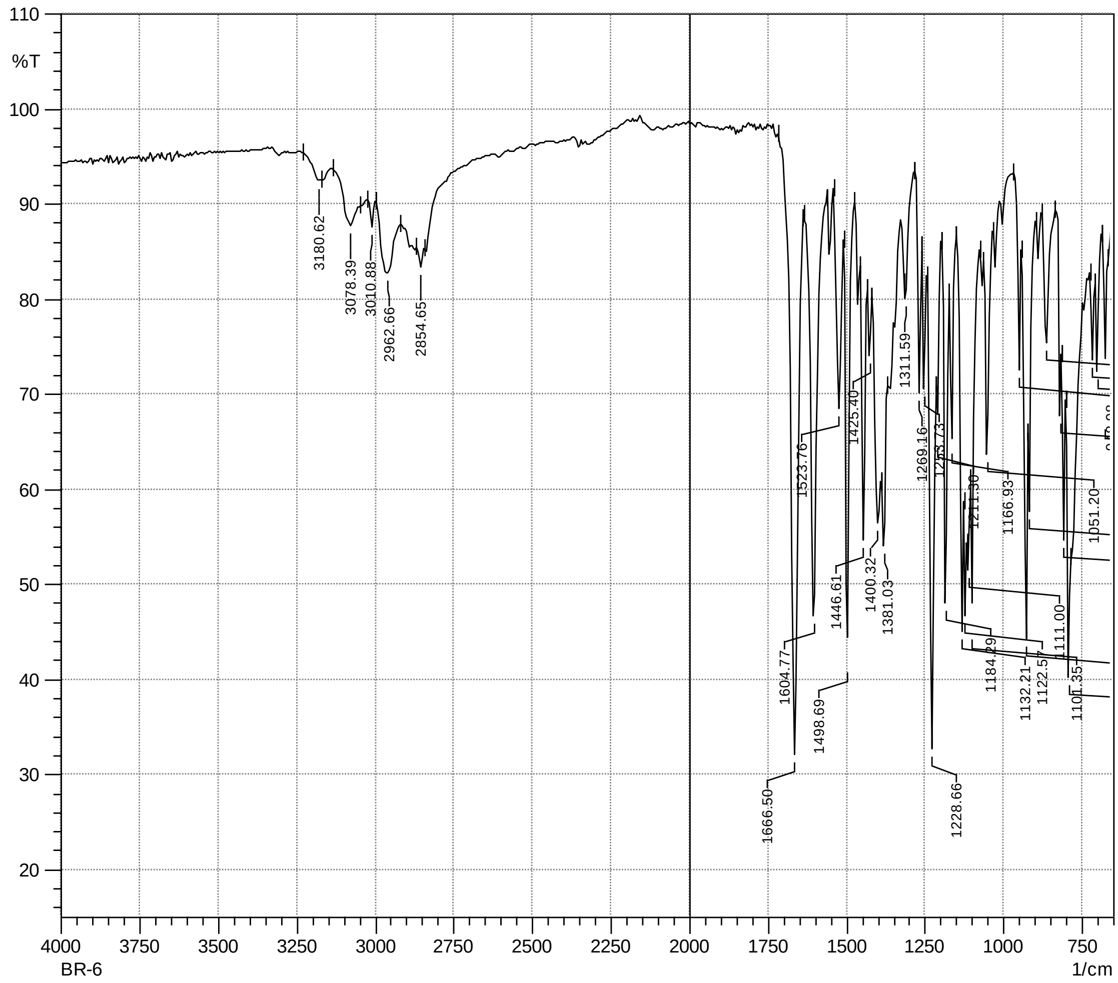

Figure S30. ^{1}H NMR spectrum of compound **3f**

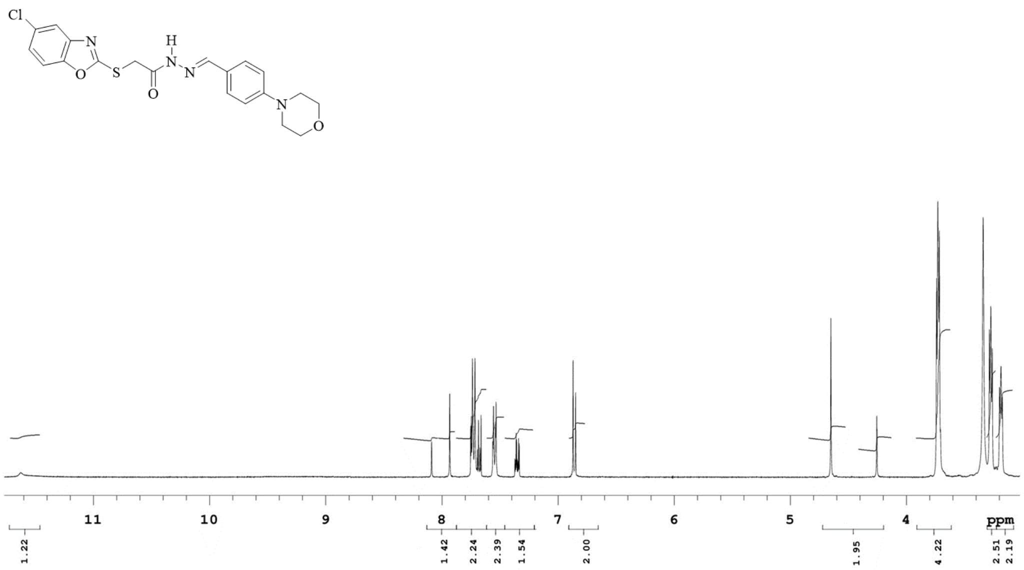

Figure S31. ^{13}C NMR spectrum of compound **3f**

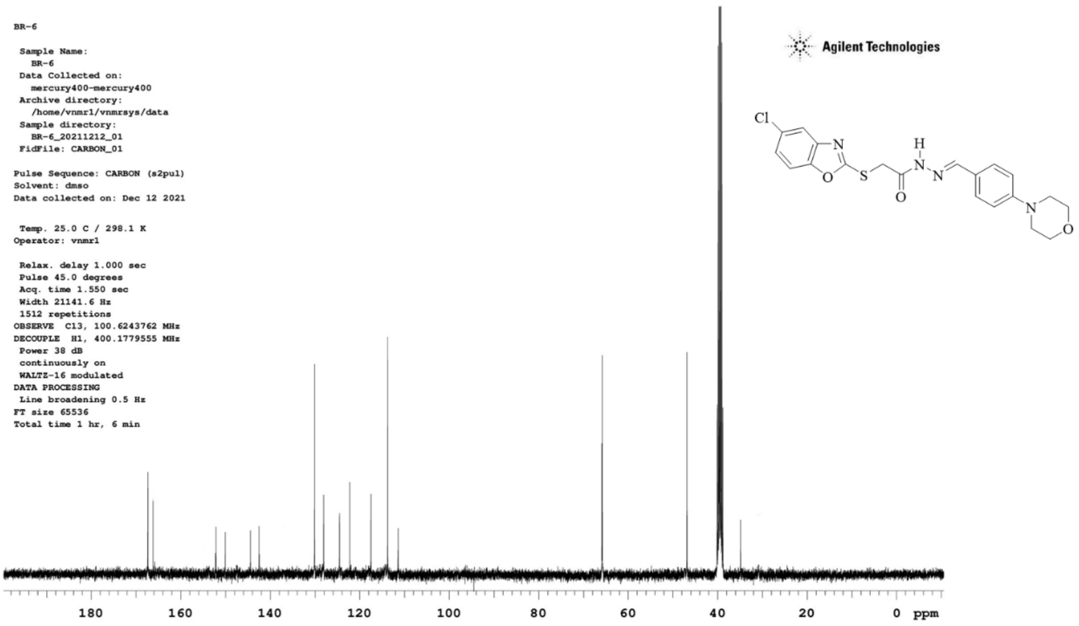

Figure S32. HRMS spectrum of compound **3f**

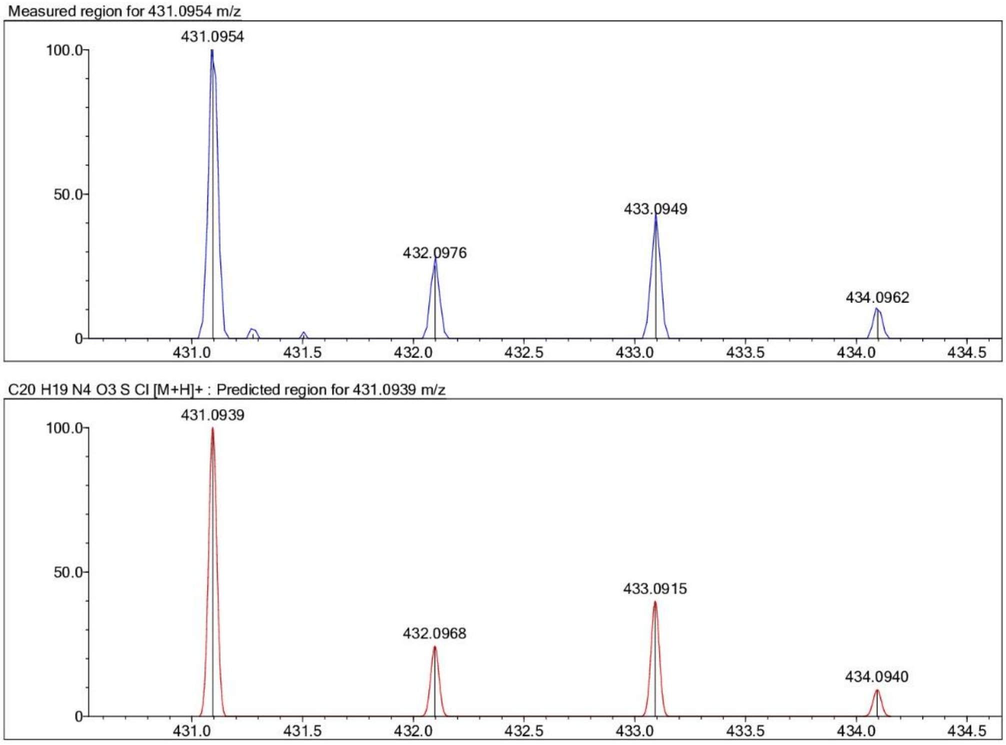

Rank	Score	Formula (M)	Ion	Meas. m/z	Pred. m/z	Df. (mDa)	Df. (ppm)	Iso	DBE
1	93.15	C20 H19 N4 O3 S Cl	[M+H]+	431.0954	431.0939	1.5	3.48	99.30	13.0

Figure S33. IR spectrum of compound **3g**

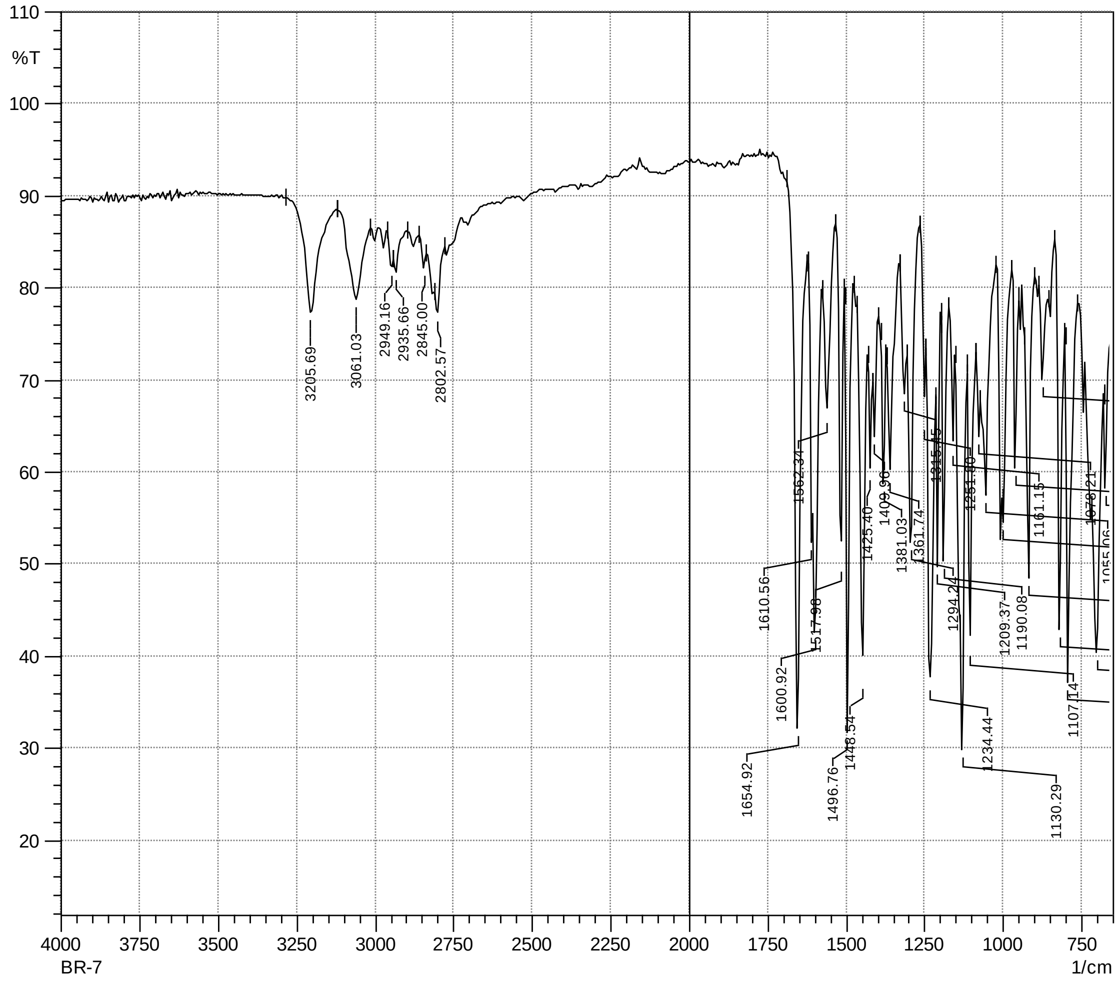

Figure S34. ^{1}H NMR spectrum of compound **3g**

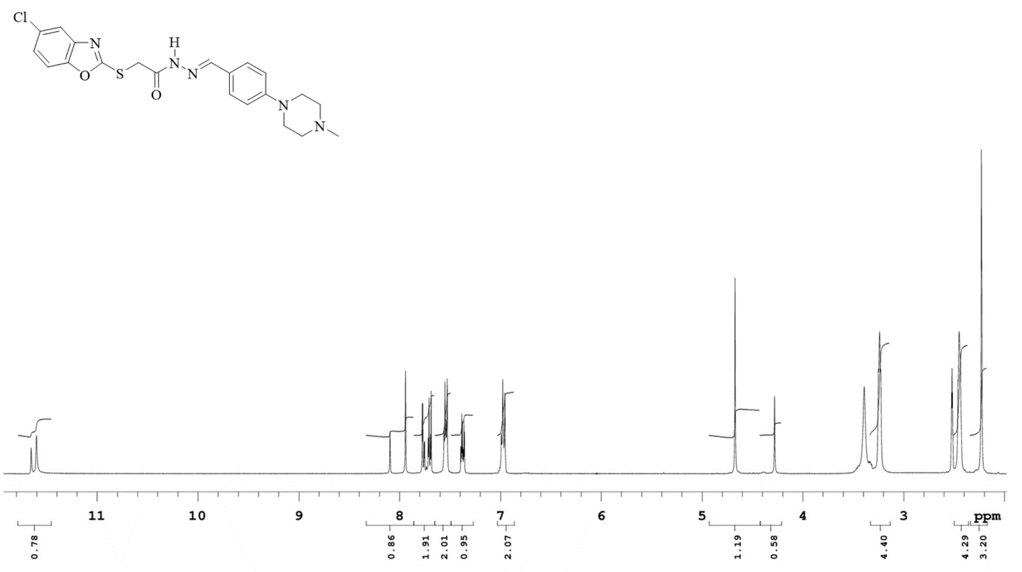

Figure S35. ^{13}C NMR spectrum of compound **3g**

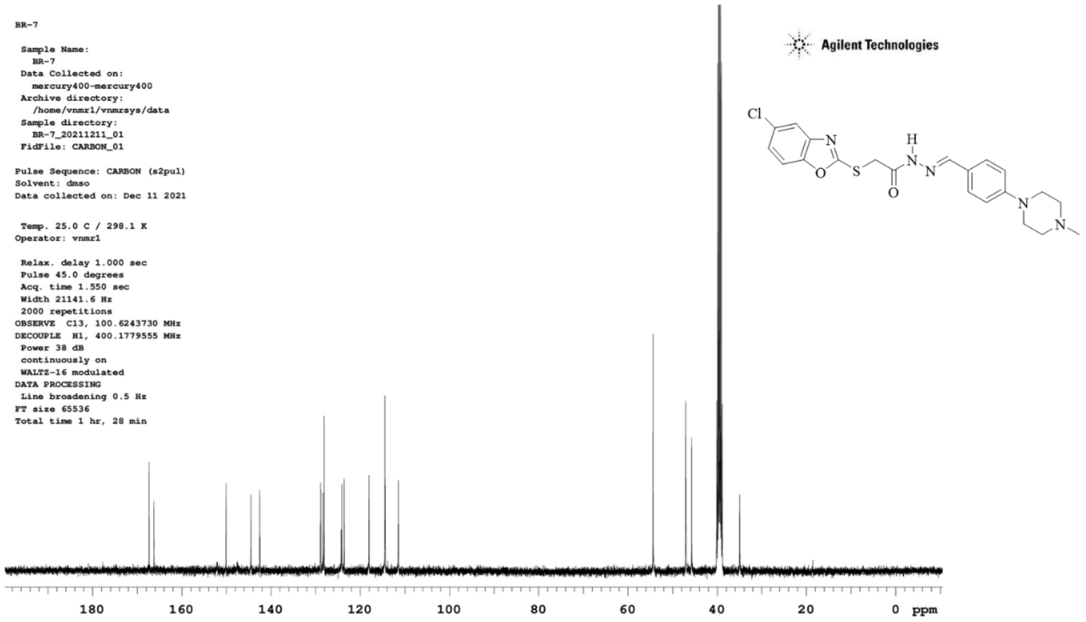

Figure S36. HRMS spectrum of compound **3g**

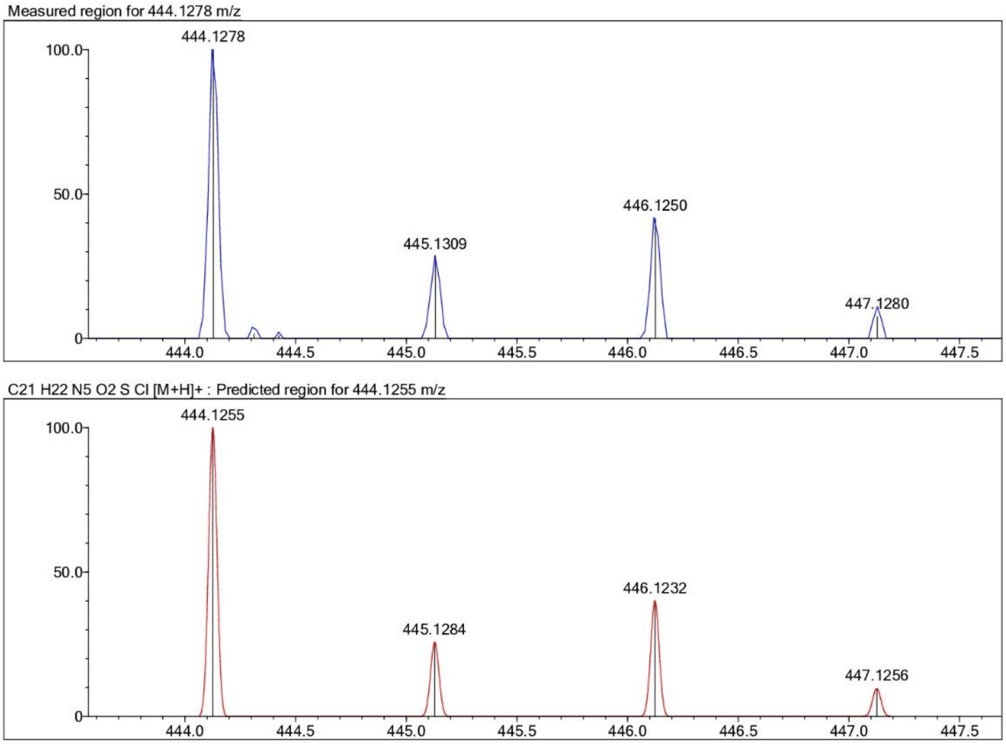

Rank	Score	Formula (M)	Ion	Meas. m/z	Pred. m/z	Df. (mDa)	Df. (ppm)	Iso	DBE
1	88.20	C21 H22 N5 O2 S Cl	[M+H]+	444.1278	444.1255	2.3	5.18	100.00	13.0

Figure S37. IR spectrum of compound **3h**

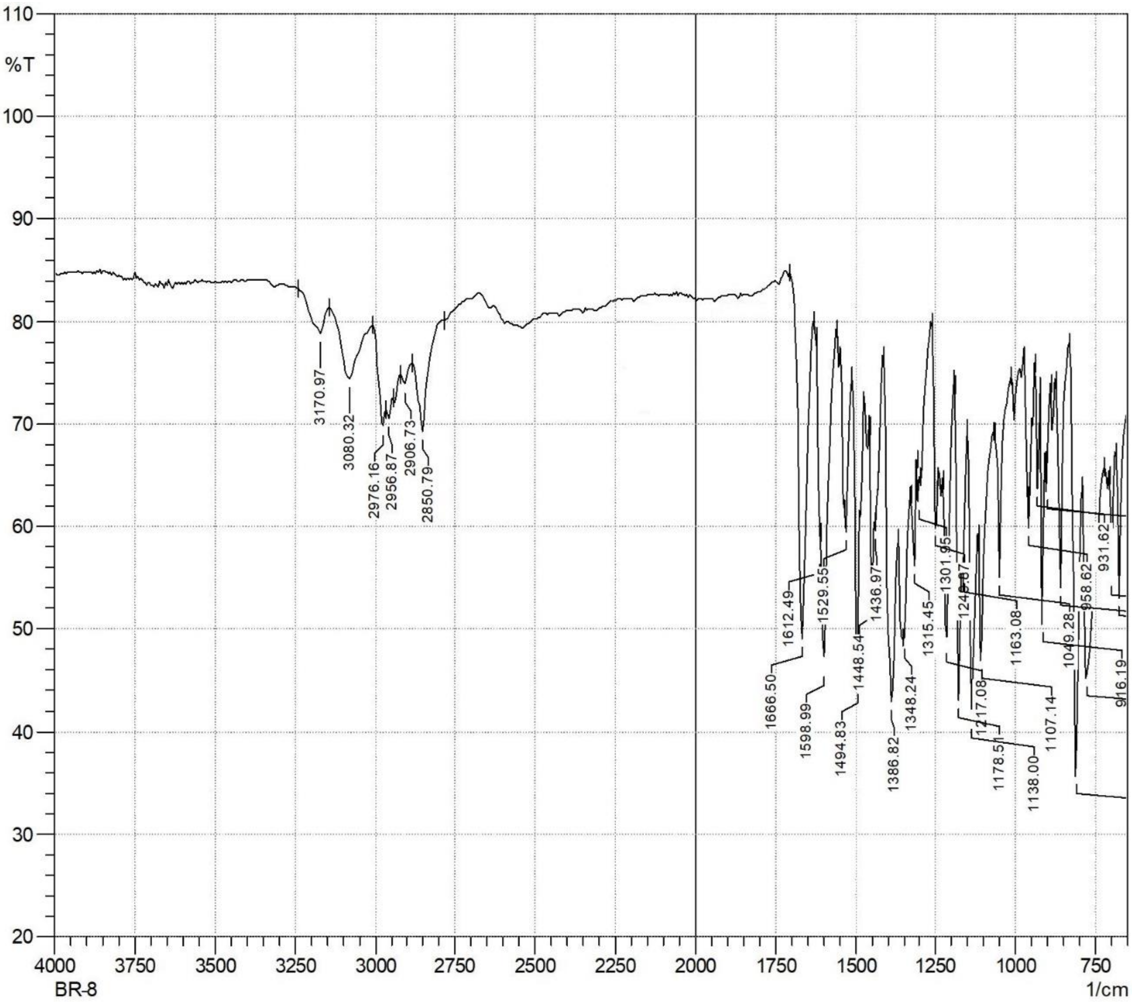

Figure S38. ^{1}H NMR spectrum of compound **3h**

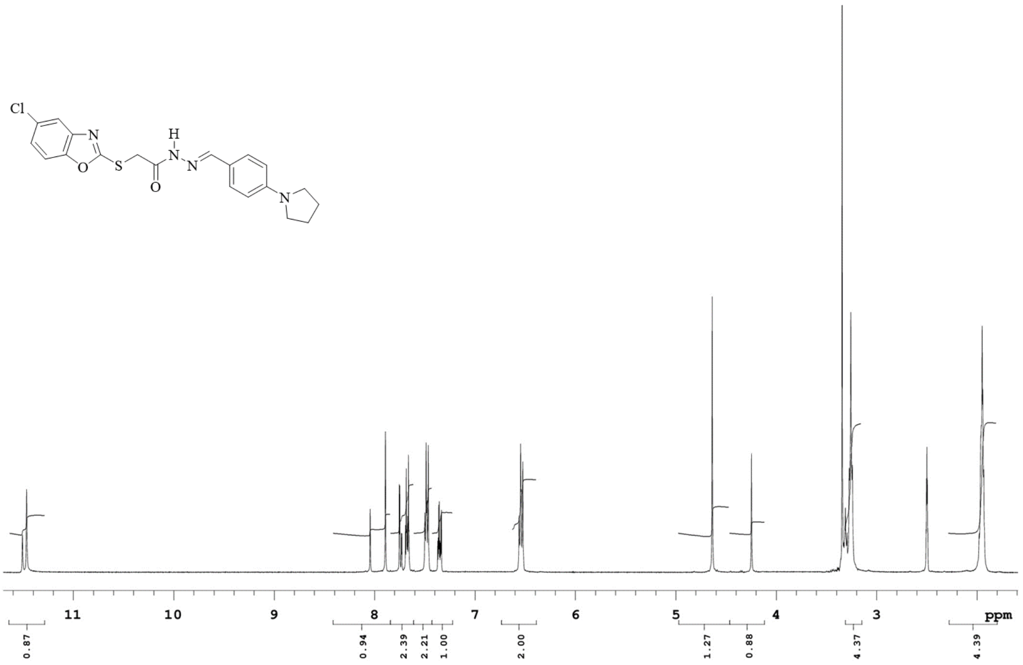

Figure S39. ^{13}C NMR spectrum of compound **3h**

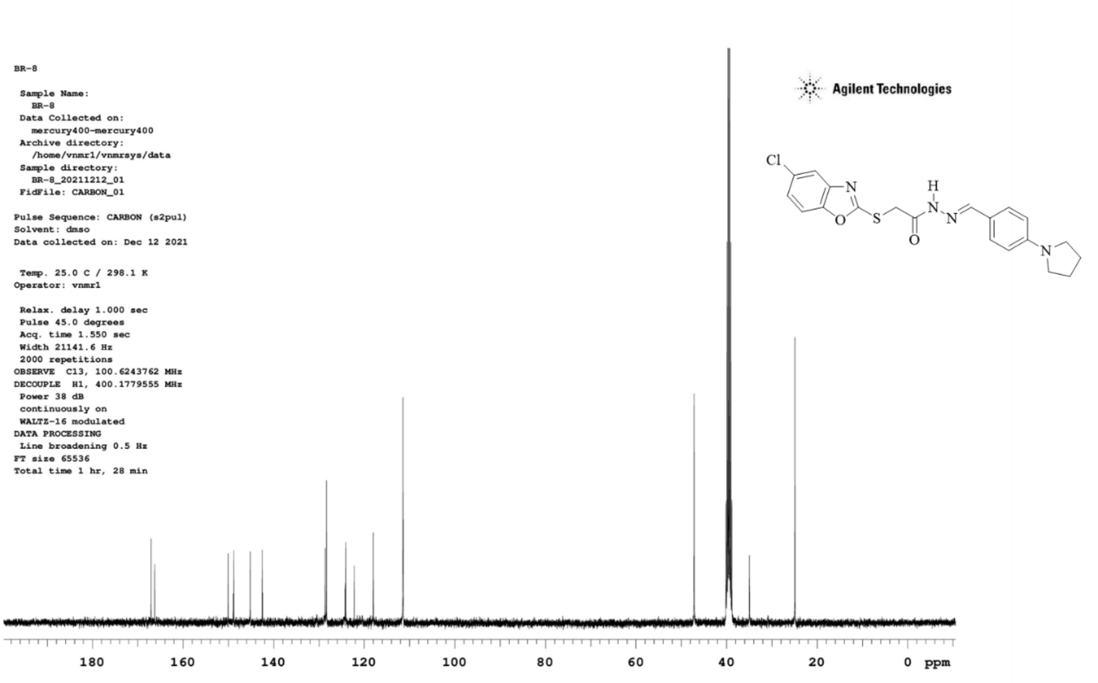

Figure S40. HRMS spectrum of compound **3h**

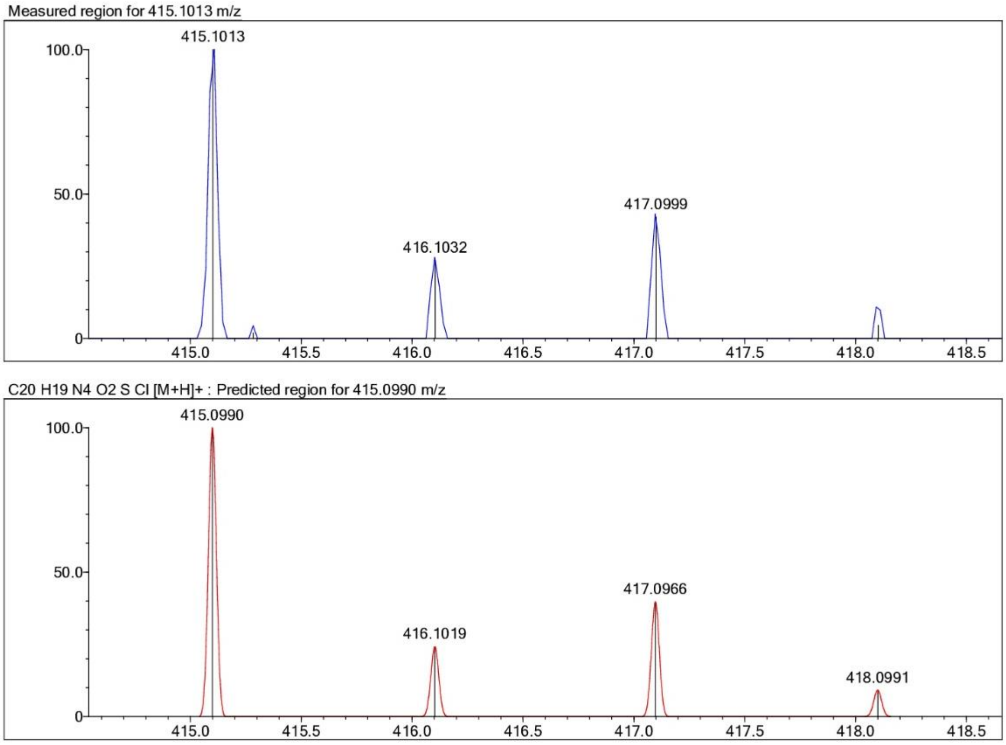

Rank	Score	Formula (M)	Ion	Meas. m/z	Pred. m/z	Df. (mDa)	Df. (ppm)	Iso	DBE
1	67.56	C20 H19 N4 O2 S Cl	[M+H]+	415.1013	415.0990	2.3	5.54	79.85	13.0

Figure S41. IR spectrum of compound **3i**

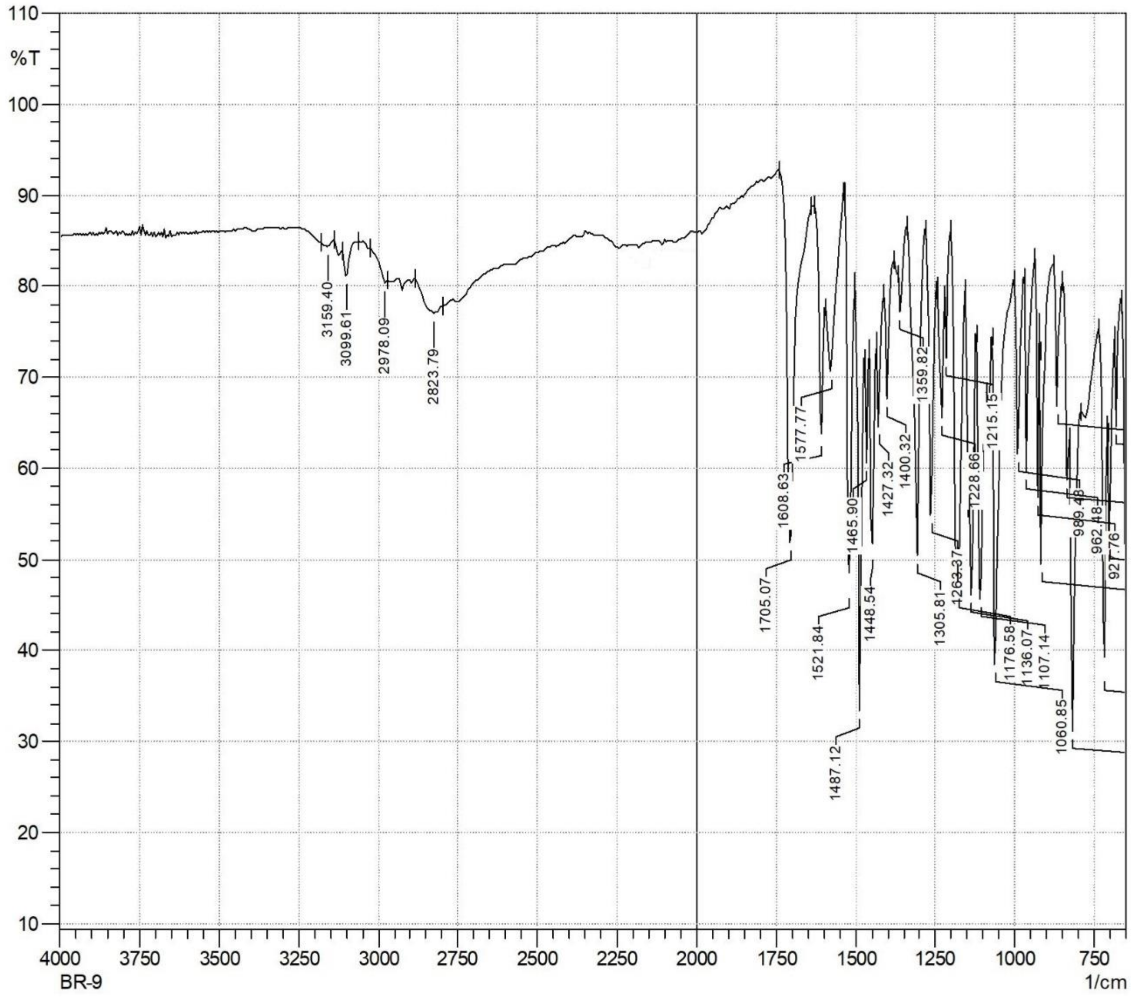

Figure S42. ^{1}H NMR spectrum of compound **3i**

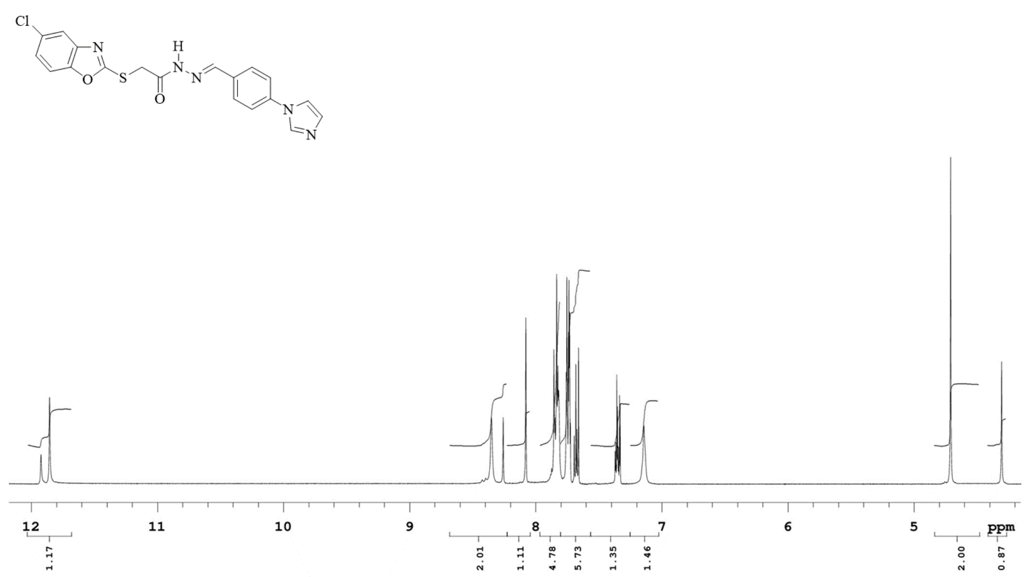

Figure S43. ^{13}C NMR spectrum of compound **3i**

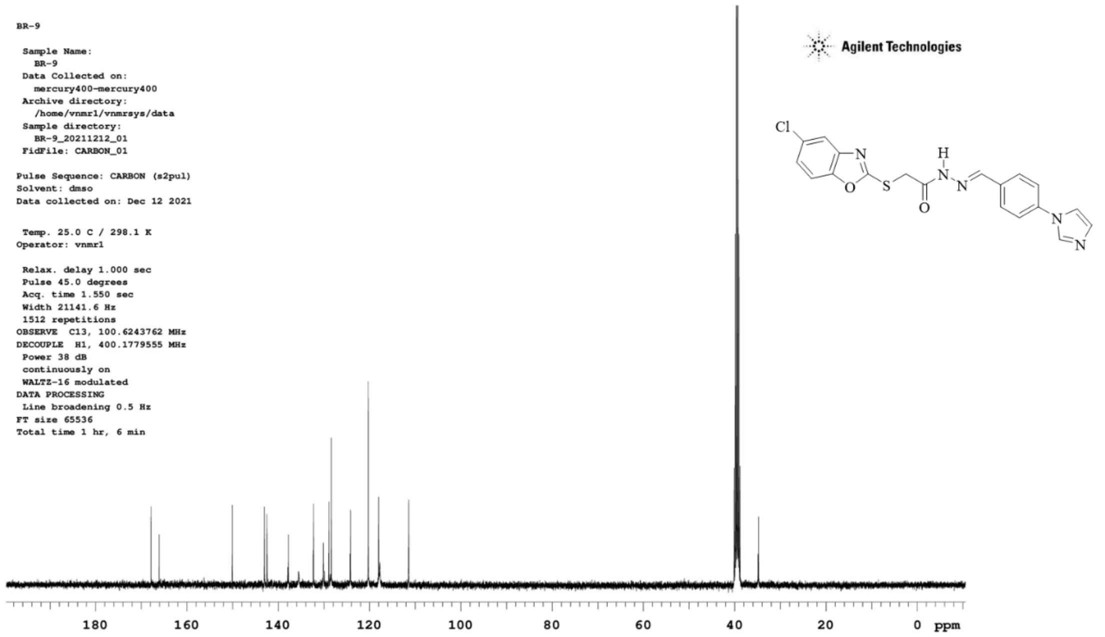

Figure S44. HRMS spectrum of compound **3i**

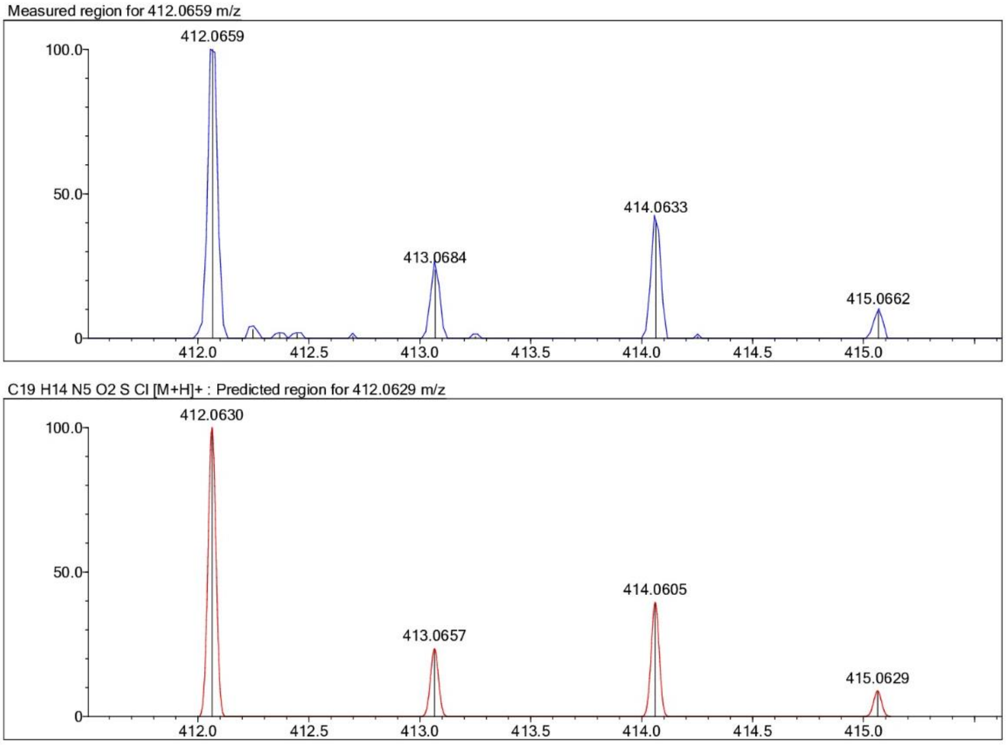

Rank	Score	Formula (M)	Ion	Meas. m/z	Pred. m/z	Df. (mDa)	Df. (ppm)	Iso	DBE
1	64.91	C19 H14 N5 O2 S Cl	[M+H]+	412.0659	412.0629	3.0	7.28	96.59	15.0

Figure S45. IR spectrum of compound **3j**

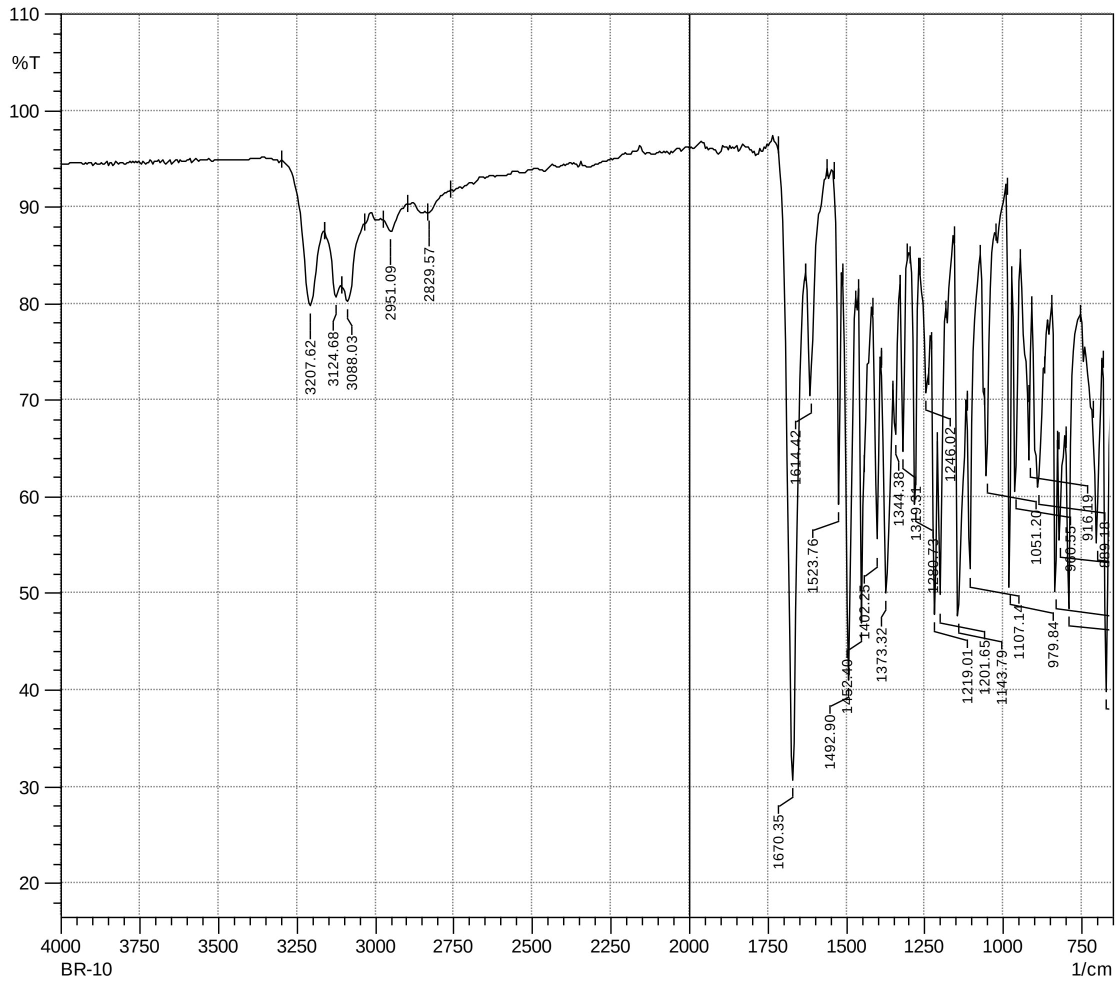

Figure S46. ^{1}H NMR spectrum of compound **3j**

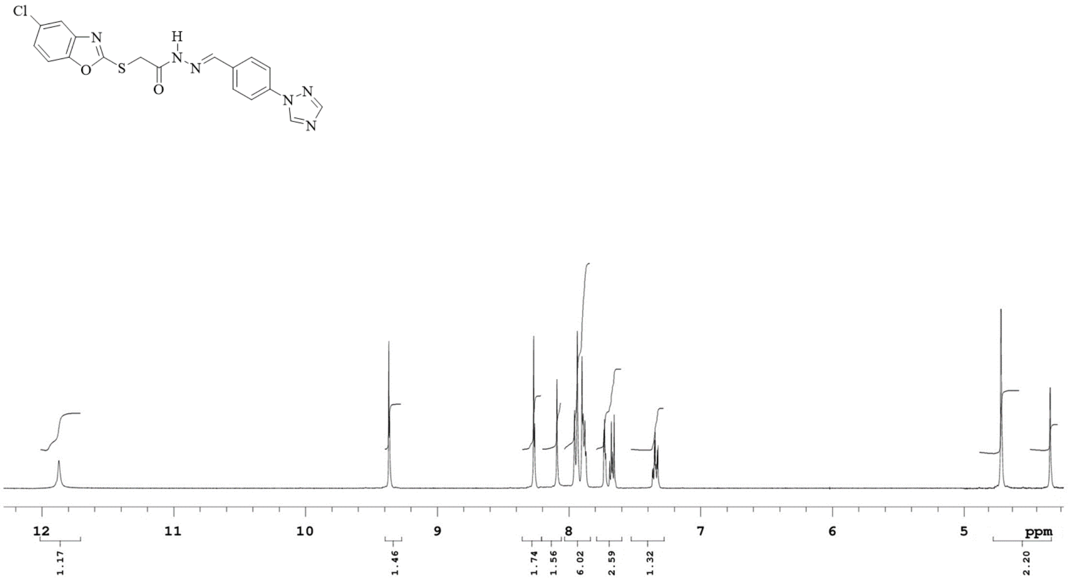

Figure S47. ^{13}C NMR spectrum of compound **3j**

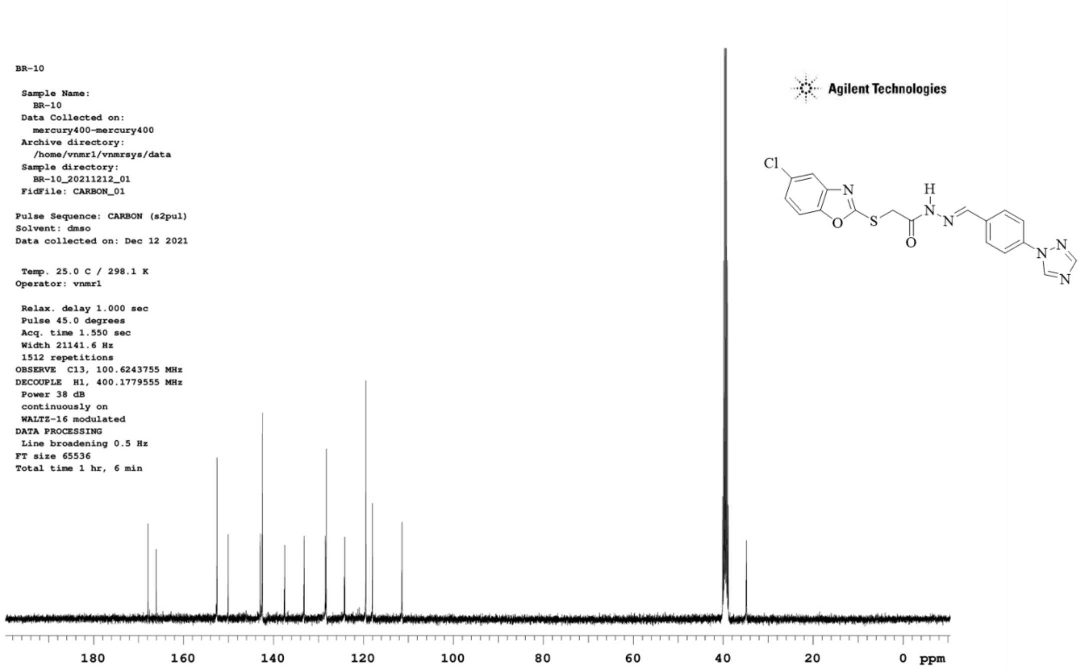

Figure S48. HRMS spectrum of compound **3j**

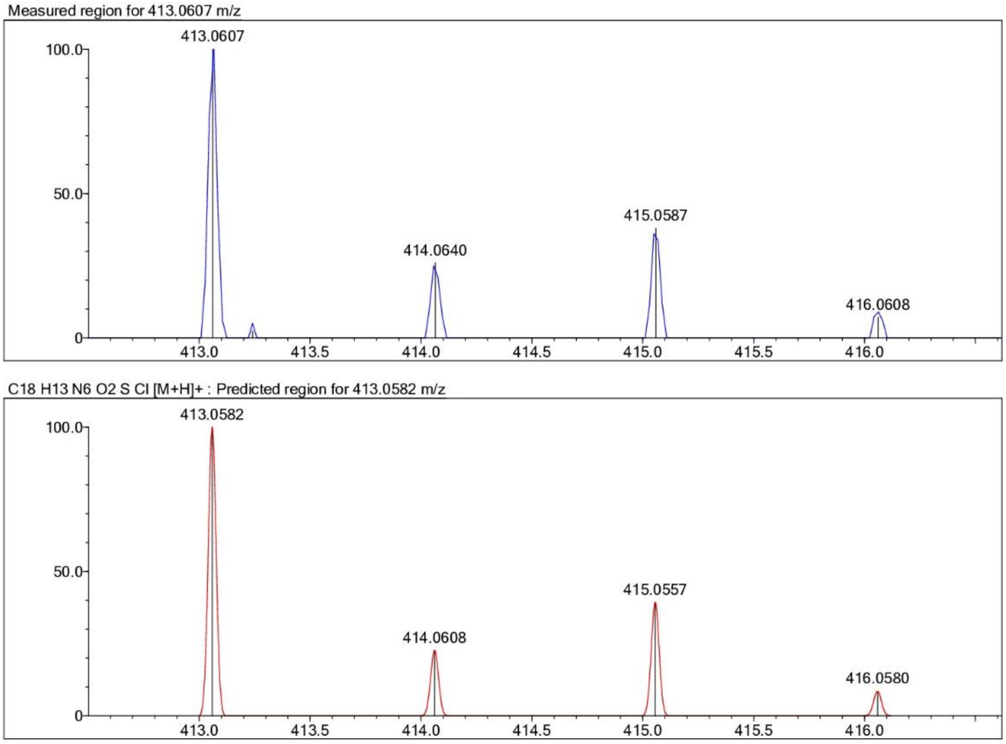

Rank	Score	Formula (M)	Ion	Meas. m/z	Pred. m/z	Df. (mDa)	Df. (ppm)	Iso	DBE
1	60.95	C18 H13 N6 O2 S Cl	[M+H]+	413.0607	413.0582	2.5	6.05	76.66	15.0
